# Supplementary material for: The mitochondrial chaperone TRAP1 regulates F-ATP synthase channel formation
Source: Cell Death Differ. 2022 May 25;29(12):2335–46. doi: 10.1038/s41418-022-01020-0 (PMC9751095; doi:10.1038/s41418-022-01020-0)
Supplement: Supplementary file 1 — Cannino et al. Supplementary information [file 41418_2022_1020_MOESM1_ESM.docx]

**Supplementary information**

**The mitochondrial chaperone TRAP1 regulates F-ATP synthase channel formation**

Giuseppe Cannino^1^, Andrea Urbani^1^, Marco Gaspari^2^, Mariaconcetta Varano^2^, Alessandro Negro^1^, Antonio Filippi^3^, Francesco Ciscato^1^, Ionica Masgras^1,4^, Christoph Gerle^5,6^, Elena Tibaldi^7^, Anna Maria Brunati^7^, Giorgio Colombo^8,9^, Giovanna Lippe^3^, Paolo Bernardi^1^, Andrea Rasola^1,*^

^1^Department of Biomedical Sciences, University of Padova, via U. Bassi 58/B, 35131, Padova, Italy

^2^Research Centre for Advanced Biochemistry and Molecular Biology, Department of Experimental and Clinical Medicine, Magna Graecia University of Catanzaro, viale Europa, 88100, Catanzaro, Italy

^3^Department of Medicine, University of Udine, via Colugna 50, 33100, Udine, Italy

^4^Institute of Neuroscience, National Research Council, Viale G. Colombo 3, 35131, Padova, Italy

^5^Institute for Protein Research, Osaka University, 3-2 Yamadaoka, Suita, Osaka, Japan

^6^RIKEN SPring-8 Center, 1-1-1 Kouto, Sayo-cho, Sayo-gun, Hyogo 679-5148, Japan

^7^Department of Molecular Medicine, University of Padova, via Gabelli 63, 35121, Padova, Italy

^8^Department of Chemistry, University of Pavia, via Taramelli 12, 27100 Pavia, Italy

^9^Institute of Chemical and Technological Sciences “Giulio Natta”– SCITEC, Via Mario Bianco 9, 20131 Milano, Italy

Correspondence should be addressed to:

Andrea Rasola ([andrea.rasola@unipd.it](mailto:andrea.rasola@unipd.it))

**Legends to Supplementary Figures**

**Supplementary Fig. 1 Identification of TRAP1 clients**. **A** NanoLC-MS/MS mass spectrometry analysis of TRAP1 interactors from four independent experiments on mitochondria from U87 cells. Only significant proteins are displayed. Criteria for significance were: FC >2 (where FC = fold-change enrichment in TRAP1 IP with respect to the control experiment) and q value < 0.05 (obtained by applying Benjamini-Hochberg correction for multiple hypothesis testing). **B** Nonyl Acridine Orange (NAO) staining of U87 cells (4 independent experiments ±SEM) indicates that TRAP1 ablation does not change mitochondrial mass.

**Supplementary Fig. 2 Binding between CyPD and OSCP. A** On the left, representative scheme of the SPR procedure followed to measure CyPD/OSCP binding. On the right, average response obtained after the injection of increase amount OSCP (1.25-25 mM) over CyPD, previously immobilized on the surface of the CM5 sensor chips. The on- and off-rate constants were by globally ﬁtting a 1:1 kinetic binding model as a function of CyPD/OSCP concentration and fitted to a Langmiur interaction model to determine K_a_. Mean values (n = 3),determined for the on-rate (K_a_ =5.9 mM). **B** Tripartite split GFP experiment on sMPNST cells in non-transfected cells stained with MitoTracker red to visualize mitochondria and check the absence of green signal (upper part), co-transfected with GFP1–9 fused to a mitochondrial import sequence, together with GFP10-SDHA and GFP11-SDHB (middle part) or with GFP10-OSCP and GFP11-SDHB (lower part). **C** Tripartite split GFP experiment on sMPNST cells co-transfected with GFP1–9 fused to a mitochondrial import sequence, together with GFP10-CyPD and GFP11-OSCP. Green dots indicate mitochondrial interaction between CyPD and OSCP. In **B** and **C**, MitoTracker (red dots) was used to stain mitochondria; superimposition of MitoTracker and GFP signal is shown as yellow dots and indicates that protein interaction occurs in mitochondria.

**Supplementary Fig. 3 Analysis of the modulation of the binding between TRAP1, CyPD and OSCP**. **A-C** Tripartite split GFP experiment on sMPNST cells co-transfected with GFP1–9 fused to a mitochondrial import sequence, together with GFP10-TRAP1 and GFP11-OSCP (**A**, cells analyzed >3820), GFP10-TRAP1 and GFP11-CyPD (**B**, cells analyzed >2145) or GFP10-CyPD and GFP11-OSCP (**C**, cells analyzed >1400). Quantification of GFP-positive cells was carried out without or with treatment with 100 µM TRAP1 inhibitor i5 (histograms in **A** and **B**), and without or with treatment with 2 µM CsA (histograms in **C**). Data are presented as mean ± SEM. ∗∗p< 0.01, ^∗∗∗^p< 0.001 with a paired two-tailed Student’s *t* test.

**Supplementary Fig. 4 Identification of multiple PTPs from F-ATP synthase and sporadic openings after TRAP1 inhibition**. **A** Full recording (10 min) of the experiment reported in Fig. 5G; currents were elicited by 3 mM Ca^2+^, after direct addition of the purified preparation of F-ATP synthase (V_cis_ = -60 mV). Discrete current levels are indicated by dotted lines. High-conductance flickering openings corresponding to up to four PTPs at full-conductance (unitary conductance 1 nS) can be observed (O1-O4), as well as openings at half-conductance (600 pS, H) and at the main sub-conductance states (200 pS, marked with a star). Red and blue dotted squares depict the portion of trace reported in Fig. 5G, corresponding to the simultaneous opening of three PTPs and flickering openings up to half-conductance. **B** A short portion (1 s) of the same experiment is reported at a more expanded scale to illustrate more in detail the complex channel activity, characterized by flickering and single-channel openings at current values corresponding to one (1.0-1.3 nS) and two (2 nS) PTPs. **C** Only sporadic channel openings could be detected after TRAP1 addition (molar ratio with F-ATP synthase of 1:1), that led to almost total current suppression (see Fig. 5H for a representative current portion). Here are reported the only noticeable channel openings (up to ~250 pS) in the 10 min recording relative to Fig. 5H. The prevalent conductance levels are marked with dotted lines and the conductance values are indicated. All along the Figure, the closed state of the channel is denoted with C.

**Supplementary Fig. 5 Partial TRAP1 inhibition of high-conductance F-ATP synthase channel activity in the presence of CyPD**. Current traces were recorded after direct incorporation of F-ATP synthase in the planar lipid bilayer and addition of 3 mM Ca^2+^; V_cis_ = –60 mV. After channel activity was detected, CyPD (molar ratio with F-ATP synthase of 2:1) was added, as shown in Fig. 6A-D. High conductance, flickering channel activity was detected in the presence of 3 mM Ca^2+^, and CyPD increased channel activity to conductance values compatible with two full conductance PTPs. Overall, TRAP1 addition (molar ratio with F-ATP synthase of 1:1 and 2:1), reduced channel activity, with remaining high-conductance openings of multiple PTPs reported here. Please note the different scales of the two current traces. The prevalent conductance levels are marked with dotted lines and the conductance values are reported; the closed state of the channel is denoted with C.

**Supplementary Table 1**. List of F-ATP synthase subunits or regulators identified as TRAP1 interactors with an unbiased nanoLC-MS/MS mass spectrometry analysis on human U87 glioblastoma cells.

FC: fold-change enrichment in TRAP1 IP with respect to control. The last three columns report, respectively, the number of unique peptides, the total number of peptides and the total peptide-spectrum matches assigned to each protein group.

| Accession | Description | Gene | FC | q value | Score | Seq. Coverage (%) | # Unique Peps | # Peps | # PSM |
| --- | --- | --- | --- | --- | --- | --- | --- | --- | --- |
| Q12931-2 | Heat shock protein 75 kDa, mitochondrial | TRAP1 | 6.39 | 0.00001 | 496 | 51.61 | 29 | 30 | 175 |
| P25705 | ATP synthase subunit alpha, mitochondrial | ATP5A1 | 2.77 | 0.00109 | 149 | 30.74 | 16 | 17 | 54 |
| P06576 | ATP synthase subunit beta, mitochondrial | ATP5B | 3.31 | 0.0005 | 73 | 22.50 | 9 | 9 | 29 |
| P36542-2 | ATP synthase subunit gamma, mitochondrial | ATP5C1 | 2.05 | 0.00278 | 53 | 23.91 | 8 | 8 | 25 |
| P48047 | ATP synthase subunit O, mitochondrial | ATP5O | 2.30 | 0.00433 | 20 | 25.35 | 6 | 6 | 10 |
| P30405 | Peptidyl-prolyl cis-trans isomerase F, mitochondrial | PPIF | 2.52 | 0.00267 | 11 | 13.53 | 3 | 4 | 9 |
| P24539 | ATP synthase F(0) complex subunit B1, mitochondrial | ATP5F1 | 3.00 | 0.00194 | 18 | 9.38 | 3 | 3 | 8 |
| O75964 | ATP synthase subunit g, mitochondrial | ATP5L | 3.03 | 0.00761 | 3 | 23.30 | 2 | 2 | 2 |
| O75947-2 | ATP synthase subunit d, mitochondrial | ATP5PD | 2.43 | 0.00313 | 8 | 18.25 | 3 | 3 | 4 |

**Supplementary Table 2**. Full list of TRAP1 interactors identified by unbiased nanoLC-MS/MS mass spectrometry analysis on human U87 glioblastoma cells.

FC: fold-change enrichment in TRAP1 IP with respect to control. The last three columns report, respectively, the number of unique peptides, the total number of peptides and the total peptide-spectrum matches assigned to each protein group.

| **Accession** | **Description** | **Gene** | **FC** | **P value** | **q value** | **Score** | **Seq Coverage** | **Unique Peptides** | **Peptides** | **PSMs** |
| --- | --- | --- | --- | --- | --- | --- | --- | --- | --- | --- |
| **P10809** | 60 kDa heat shock protein | **HSPD1** | 5,79 | 0,00001 | 0,0005 | 177 | 37,87 | 19 | 19 | 70 |
| **Q9UJZ1-2** | Stomatin-like protein 2 | **STOML2** | 4,25 | 0,00001 | 0,0005 | 33 | 21,86 | 5 | 5 | 12 |
| **Q96RP9-2** | Elongation factor G | **GFM1** | 4,18 | 0,00024 | 0,00125 | 6 | 4,16 | 3 | 3 | 4 |
| **Q8TCS8** | Polyrib Nucleotidyltransf 1 | **PNPT1** | 3,49 | 0,00008 | 0,00081 | 10 | 5,75 | 5 | 5 | 5 |
| **P11177-2** | Pyr dehyd E1 sub beta | **PDHB** | 3,47 | 0,00026 | 0,00129 | 13 | 11,14 | 4 | 4 | 7 |
| **P21796** | Porin 31HL | **VDAC1** | 3,44 | 0,00003 | 0,0007 | 51 | 27,92 | 6 | 7 | 19 |
| **Q6YN16-2** | Hydroxysteroid deh | **HSDL2** | 3,43 | 0,00145 | 0,00364 | 2 | 5,51 | 2 | 2 | 2 |
| **O95202** | Proton/calcium exchanger | **LETM1** | 3,43 | 0,00005 | 0,0007 | 26 | 12,99 | 9 | 9 | 12 |
| **P36776-2** | Lon protease homolog | **LONP1** | 3,43 | 0,00004 | 0,0007 | 48 | 13,3 | 13 | 13 | 19 |
| **P49748-2** | VLCAD | **ACADVL** | 3,42 | 0,00003 | 0,0007 | 9 | 6,79 | 4 | 4 | 4 |
| **P05141** | ADP/ATP translocase 2 | **SLC25A5** | 3,34 | 0,00015 | 0,00098 | 34 | 17,45 | 6 | 6 | 16 |
| **O95203** | EF-hand dom-cont-prot 1 | **LETM1** | 3,34 | 0,00014 | 0,00098 | 26 | 14,35 | 3,5 | 3,5 | 12 |
| **Q16836** | Hydroxyacyl-coenzymeA-deh | **HADH** | 3,33 | 0,0001 | 0,00081 | 12 | 9,24 | 5 | 5 | 6 |
| **P30048-2** | Antioxidant protein 1 | **PRDX3** | 3,33 | 0,00005 | 0,0007 | 11 | 7,14 | 2 | 2 | 4 |
| **P06576** | ATP synthase subunit beta | **ATP5F1B** | 3,31 | 0,00001 | 0,0005 | 73 | 22,5 | 9 | 9 | 29 |
| **Q9Y3D9** | 28S ribosomal protein S23 | **MRPS23** | 3,29 | 0,00011 | 0,00087 | 16 | 13,68 | 2 | 2 | 4 |
| **O94826** | Translocase outer-mem70kDa | **TOMM70** | 3,27 | 0,00001 | 0,0005 | 14 | 6,09 | 5 | 5 | 7 |
| **P36957** | OGDC-E2 **DLST** | | 3,26 | 0,00002 | 0,0007 | 56 | 16,34 | 8 | 8 | 21 |
| **O76031** | Clp protease sub | **CLPX** | 3,15 | 0,00003 | 0,0007 | 3 | 3,16 | 2 | 2 | 2 |
| **O75746-2** | Aspartate glutamate carrier 1 | **SLC25A12** | 3,14 | 0,00001 | 0,0005 | 10 | 5,25 | 3 | 3 | 4 |
| **P11310** | MCAD | **ACADM** | 3,12 | 0,00001 | 0,0005 | 10 | 6,41 | 3 | 3 | 4 |
| **P54886-2** | D-1-pyrroline-5-carb- synth] | **ALDH18A1** | 3,10 | 0,00039 | 0,00149 | 17 | 5,67 | 5 | 5 | 7 |
| **P41250** | Glycine--tRNA ligase | **GARS1** | 3,09 | 0,0003 | 0,00138 | 2 | 2,03 | 2 | 2 | 2 |
| **P49411** | Elongation factor Tu | **TUFM** | 3,08 | 0,00011 | 0,00087 | 43 | 25,22 | 11 | 11 | 16 |
| **Q16891-2** | MICOS complex sub MIC60 | **IMMT** | 3,07 | 0,00011 | 0,00087 | 95 | 23,03 | 20 | 20 | 39 |
| **Q3ZCQ8** | Inner membr transl TIM50 | **TIMM50** | 3,05 | 0,00192 | 0,00438 | 16 | 5,67 | 3 | 3 | 5 |
| **Q9H2U2-3** | Inorganic pyrophosphatase 2 | **PPA2** | 3,03 | 0,00035 | 0,00147 | 9 | 5,9 | 2 | 2 | 3 |
| **P38646** | Stress-70 protein | **HSPA9** | 3,03 | 0,00024 | 0,00125 | 240 | 39,91 | 27 | 28 | 103 |
| **O75964** | ATP synthase subunit g | **ATP5MG** | 3,03 | 0,00412 | 0,00761 | 4 | 23,3 | 2 | 2 | 2 |
| **P24539** | ATP synthase subunit b | **ATP5PB** | 3,00 | 0,00061 | 0,00194 | 18 | 9,38 | 3 | 3 | 8 |
| **Q9HAV7** | GrpE protein homolog 1 | **GRPEL1** | 2,98 | 0,0001 | 0,00087 | 8 | 12,44 | 3 | 3 | 4 |
| **P19367-4** | Hexokinase-1 | **HK1** | 2,94 | 0,00004 | 0,0007 | 75 | 17,68 | 17 | 17 | 32 |
| **Q9NSE4** | Isoleucine--tRNA ligase | **IARS2** | 2,91 | 0,00006 | 0,00071 | 5 | 2,08 | 2 | 2 | 2 |
| **P24752** | Acetyl-CoA acetyltransferase | **ACAT1** | 2,91 | 0,00015 | 0,00098 | 17 | 9,6 | 4 | 4 | 6 |
| **P07954-2** | Fumarate hydratase | **FH** | 2,91 | 0,00001 | 0,0005 | 29 | 17,77 | 8 | 8 | 10 |
| **O94925-2** | Glutaminase Isoform 2 | **GLS** | 2,88 | 0,00043 | 0,00154 | 6 | 14,2 | 2 | 2 | 3 |
| **P42704** | 130 kDa leucine-rich protein | **LRPPRC** | 2,87 | 0,00008 | 0,00081 | 52 | 13,63 | 22 | 22 | 25 |
| **Q96I99** | Succinyl-CoA synth beta-G | **SUCLG2** | 2,81 | 0,00011 | 0,00087 | 16 | 9,49 | 4 | 4 | 6 |
| **P31930** | Complex III subunit 1 | **UQCRC1** | 2,80 | 0,00009 | 0,00081 | 13 | 8,54 | 4 | 4 | 5 |
| **P25705** | ATP synthase subunit alpha | **ATP5F1A** | 2,77 | 0,0002 | 0,00109 | 150 | 30,74 | 16 | 17 | 54 |
| **Q6UB35** | Formyltetrahydrofolate synth | **MTHFD1L** | 2,74 | 0,0004 | 0,00151 | 9 | 4,81 | 6 | 6 | 7 |
| **P28331-3** | Complex I-75kD | **NDUFS1** | 2,73 | 0,00017 | 0,00102 | 7 | 6,01 | 4 | 4 | 4 |
| **P45880-2** | Outer-mit memb prot porin 2 | **VDAC2** | 2,72 | 0,00027 | 0,00132 | 23 | 26,86 | 6 | 7 | 12 |
| **O96008-2** | Transl outer memb 40 kDasub | **TOMM40** | 2,70 | 0,00177 | 0,00419 | 5 | 6,08 | 2 | 2 | 3 |
| **P13995** | Methenyltetrahydrofolate Bif | **MTHFD2** | 2,70 | 0,00008 | 0,00081 | 8 | 8,57 | 3 | 3 | 4 |
| **Q00325-2** | Phosphate carrier | **SLC25A3** | 2,67 | 0,00005 | 0,0007 | 25 | 9,7 | 4 | 4 | 10 |
| **P21912** | Iron-sulfur sub of complex II | **SDHB** | 2,67 | 0,00036 | 0,00148 | 7 | 7,14 | 3 | 3 | 4 |
| **Q9NVT9** | Armadillo repeat-cont prot 1 | **ARMC1** | 2,64 | 0,00037 | 0,00148 | 2 | 7,8 | 3 | 3 | 3 |
| **P55084** | Trifunctional enz sub beta | **HADHB** | 2,64 | 0,00008 | 0,00081 | 27 | 17,51 | 8 | 8 | 12 |
| **P40939** | Trifunctional enz sub alpha | **HADHA** | 2,62 | 0,00007 | 0,00077 | 32 | 12,84 | 9 | 9 | 15 |
| **Q02218-2** | 2-oxoglutarate dehydr | **OGDH** | 2,61 | 0,00065 | 0,002 | 8 | 2,36 | 3 | 3 | 3 |
| **Q9H9B4** | Sideroflexin-1 | **SFXN1** | 2,60 | 0,00009 | 0,00081 | 12 | 18,01 | 5 | 5 | 5 |
| **Accession** | **Description** | **Gene** | **FC** | **P value** | **q value** | **Score** | **Seq Coverage** | **Unique Peptides** | **Peptides** | **PSMs** |
| **O43615** | Import inner-memb transl | **TIMM44** | 2,55 | 0,00016 | 0,00102 | 14 | 11,95 | 5 | 5 | 6 |
| **P08559-3** | Pyr dehy E1 comp sub alpha | **PDHA1** | 2,53 | 0,00012 | 0,00089 | 10 | 7,52 | 3 | 3 | 4 |
| **P30405** | Cyclophilin D | **PPIF** | 2,52 | 0,00095 | 0,00267 | 11 | 13,53 | 3 | 4 | 9 |
| **P35232** | Prohibitin | **PHB** | 2,51 | 0,00041 | 0,00154 | 27 | 20,96 | 6 | 6 | 11 |
| **O96000-2** | Complex I-PDSW sub 10 | **NDUFB10** | 2,49 | 0,00005 | 0,0007 | 9 | 12,5 | 2 | 2 | 4 |
| **O75390** | Citrate synthase | **CS** | 2,48 | 0,0004 | 0,00151 | 21 | 10,94 | 5 | 5 | 9 |
| **P13804** | Alpha-ETF | **ETFA** | 2,44 | 0,00062 | 0,00194 | 8 | 12,91 | 4 | 4 | 6 |
| **Q99714-2** | Hydroxyacyl-CoA dehyd -2 | **HSD17B10** | 2,43 | 0,00024 | 0,00125 | 25 | 26,19 | 5 | 5 | 11 |
| **O75947-2** | ATP synthase subunit d | **ATP5PD** | 2,43 | 0,00118 | 0,00313 | 9 | 18,25 | 3 | 3 | 4 |
| **Q9NVI7-3** | ATPase family AAA Isoform 3 | **ATAD3A** | 2,42 | 0,00145 | 0,00364 | 19 | 9,27 | 6 | 6 | 9 |
| **O60313** | Optic atrophy protein 1 | **OPA1** | 2,41 | 0,00035 | 0,00147 | 19 | 5,52 | 5 | 5 | 7 |
| **Q969Z0-2** | Protein TBRG4 isoform 2 | **TBRG4** | 2,40 | 0,00255 | 0,00531 | 4 | 3,45 | 2 | 2 | 2 |
| **Q00059** | Transcription factor A | **TFAM** | 2,39 | 0,01113 | 0,01647 | 15 | 14,23 | 4 | 4 | 5 |
| **P27144** | Adenylate kinase 4 | **AK4** | 2,36 | 0,00043 | 0,00154 | 7 | 9,42 | 2 | 2 | 3 |
| **Q9GZT3-2** | SRA stem-loop-interRNAbp | **SLIRP** | 2,34 | 0,00468 | 0,00831 | 24 | 36,45 | 4 | 4 | 8 |
| **Q92665** | 28S ribosomal protein S31 | **MRPS31** | 2,34 | 0,00073 | 0,0022 | 6 | 8,1 | 3 | 3 | 3 |
| **O95831-3** | Apoptosis-inducing factor 1 | **AIFM1** | 2,32 | 0,0046 | 0,00827 | 20 | 13,14 | 7 | 7 | 8 |
| **P38117** | Beta-ETF | **ETFB** | 2,32 | 0,00025 | 0,00129 | 24 | 23,14 | 6 | 6 | 10 |
| **Q5JRX3-3** | Presequence protease Is 3 | **PITRM1** | 2,32 | 0,00038 | 0,00148 | 11 | 4,15 | 5 | 5 | 6 |
| **Q8N983-4** | 39S ribosomal protein L43 | **MRPL43** | 2,32 | 0,00199 | 0,00453 | 9 | 15,72 | 3 | 3 | 4 |
| **P09669** | Cytochrome c oxidase sub 6C | **COX6C** | 2,31 | 0,00053 | 0,00175 | 8 | 17,33 | 2 | 2 | 5 |
| **P48047** | ATP synthase subunit O | **ATP5PO** | 2,30 | 0,00187 | 0,00433 | 20 | 25,35 | 6 | 6 | 10 |
| **Q9NX63** | MICOS complex sub MIC19 | **CHCHD3** | 2,26 | 0,00188 | 0,00433 | 20 | 16,74 | 5 | 5 | 9 |
| **P57105** | Synaptojanin-2-binding prot | **SYNJ2BP** | 2,25 | 0,0103 | 0,01552 | 5 | 15,17 | 2 | 2 | 3 |
| **P43155-3** | Carnitine O-acetyltransferase | **CRAT** | 2,25 | 0,00182 | 0,00428 | 7 | 4,23 | 2 | 2 | 2 |
| **P30084** | Enoyl-CoA hydratase | **ECHS1** | 2,25 | 0,00032 | 0,00145 | 5 | 6,21 | 2 | 2 | 2 |
| **Q99623** | Prohibitin-2 | **PHB2** | 2,25 | 0,00152 | 0,00373 | 50 | 29,43 | 10 | 10 | 20 |
| **P47985** | Complex III subunit 5 | **UQCRFS1** | 2,23 | 0,00047 | 0,00162 | 21 | 13,5 | 4 | 4 | 8 |
| **P50213** | Isocitric dehyd sub alpha | **IDH3A** | 2,22 | 0,00034 | 0,00146 | 18 | 9,02 | 4 | 4 | 8 |
| **P53597** | Succinyl-CoA synt sub alpha | **SUCLG1** | 2,20 | 0,00103 | 0,00278 | 6 | 7,23 | 2 | 2 | 2 |
| **P00387-2** | NADH-cytochrome b5 red 3 | **CYB5R3** | 2,18 | 0,02171 | 0,02913 | 8 | 8,99 | 2 | 2 | 3 |
| **P11498** | Pyruvate carboxylase | **PC** | 2,17 | 0,00255 | 0,00531 | 11 | 2,04 | 2 | 2 | 3 |
| **P22695** | Complex III subunit 2 | **UQCRC2** | 2,16 | 0,00356 | 0,00679 | 13 | 12,58 | 5 | 5 | 5 |
| **P10515** | Pyr dehydr complex comp E2 | **DLAT** | 2,16 | 0,00061 | 0,00194 | 8 | 3,86 | 3 | 3 | 4 |
| **Q99798** | Aconitate hydratase | **ACO2** | 2,16 | 0,00029 | 0,00138 | 19 | 5,51 | 4 | 4 | 7 |
| **P14618** | Pyruvate kinase PKM | **PKM** | 2,09 | 0,0042 | 0,00772 | 24 | 16,01 | 9 | 9 | 11 |
| **Q96BP2** | 28S ribosomal protein S37 | **CHCHD1** | 2,08 | 0,00481 | 0,00842 | 8 | 22,88 | 2 | 2 | 3 |
| **P10606** | Cytochrome c oxidase sub 5B | **COX5B** | 2,07 | 0,0005 | 0,00169 | 13 | 24,03 | 4 | 4 | 6 |
| **P04179-4** | Superoxide dismutase | **SOD2** | 2,06 | 0,00473 | 0,00836 | 2 | 8,52 | 2 | 2 | 2 |
| **P36542-2** | ATP synthase sub gamma | **ATP5F1C** | 2,05 | 0,00102 | 0,00278 | 54 | 23,91 | 8 | 8 | 25 |
| **Q14197** | Peptidyl-tRNA hydrolase ICT1 | **MRPL58** | 2,04 | 0,0143 | 0,02021 | 4 | 9,71 | 2 | 2 | 3 |
| **Q96HS1** | Ser/thre-prot phosp PGAM5 | **PGAM5** | 2,02 | 0,00042 | 0,00154 | 31 | 20,07 | 6 | 6 | 10 |
