# Supplementary figures and images for: The mitochondrial chaperone TRAP1 regulates F-ATP synthase channel formation
Source: Cell Death Differ. 2022 May 25;29(12):2335–46. doi: 10.1038/s41418-022-01020-0 (PMC9751095; doi:10.1038/s41418-022-01020-0)

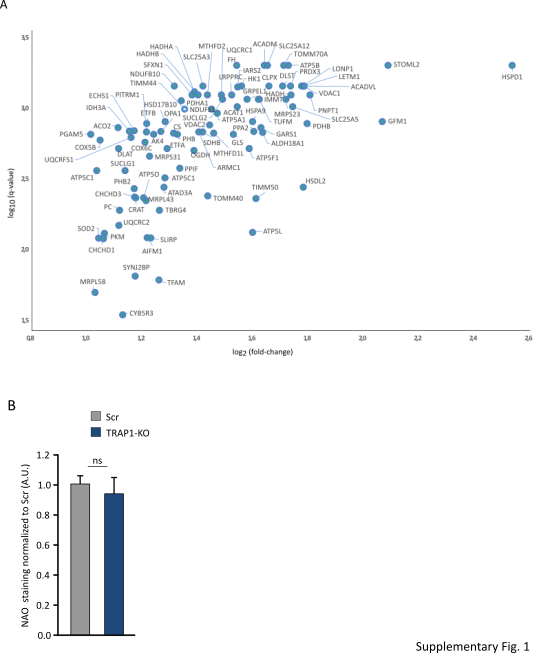

Supplement: Supplementary file 2 — Supplementary Fig. 1 [file 41418_2022_1020_MOESM2_ESM.tif]

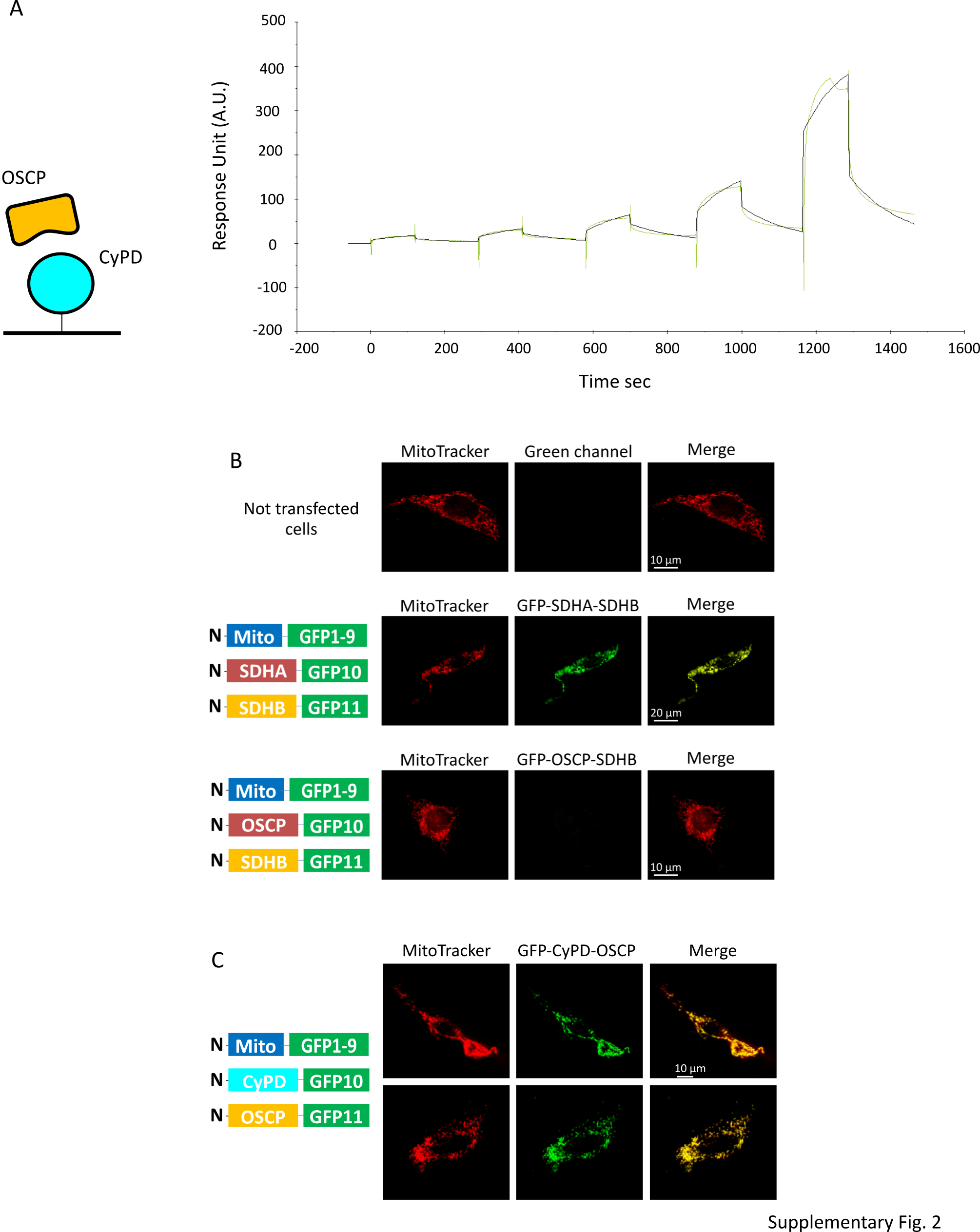

Supplement: Supplementary file 3 — Supplementary Fig. 2 [file 41418_2022_1020_MOESM3_ESM.tif]

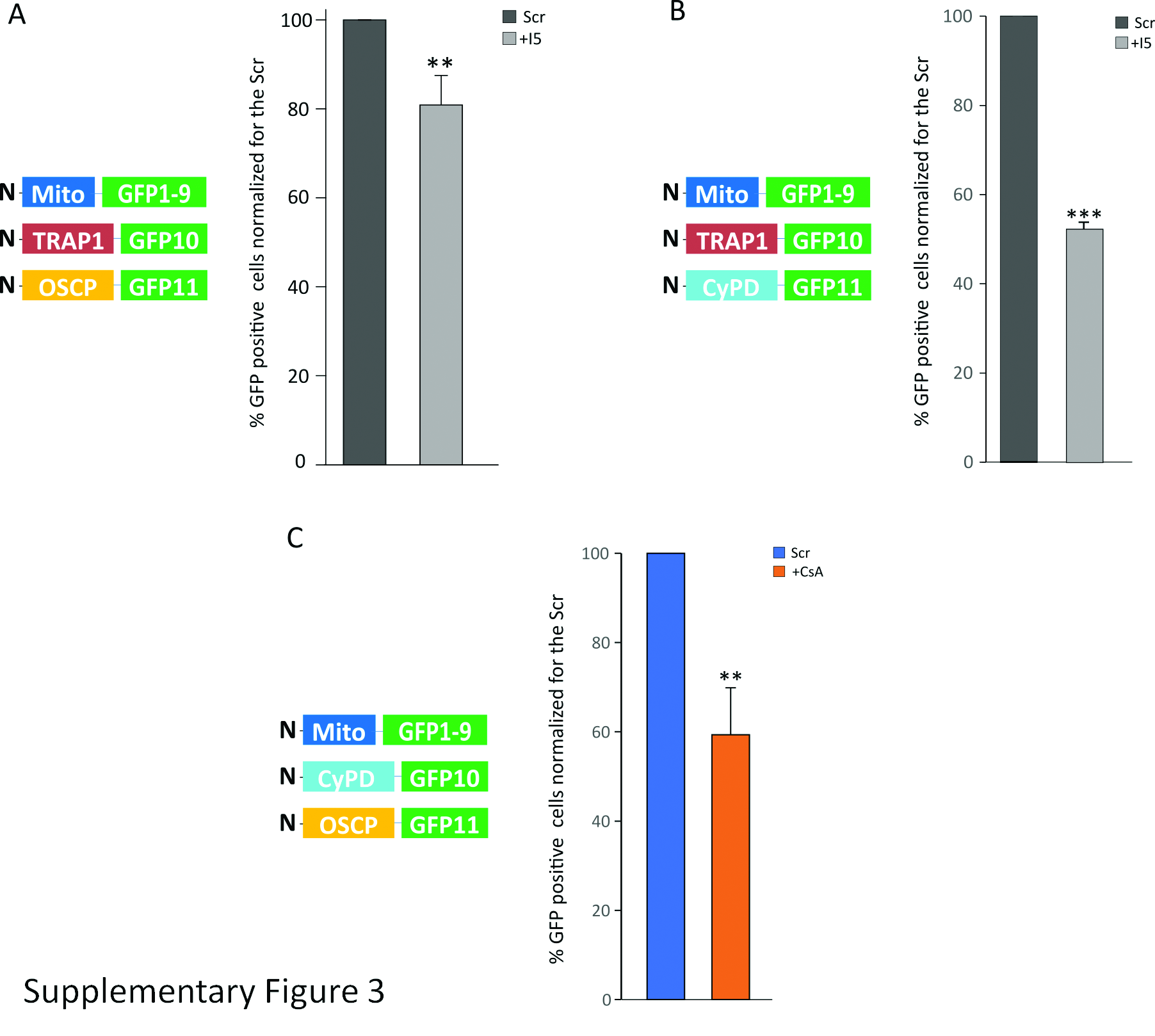

Supplement: Supplementary file 4 — Supplementary Fig. 3 [file 41418_2022_1020_MOESM4_ESM.tif]

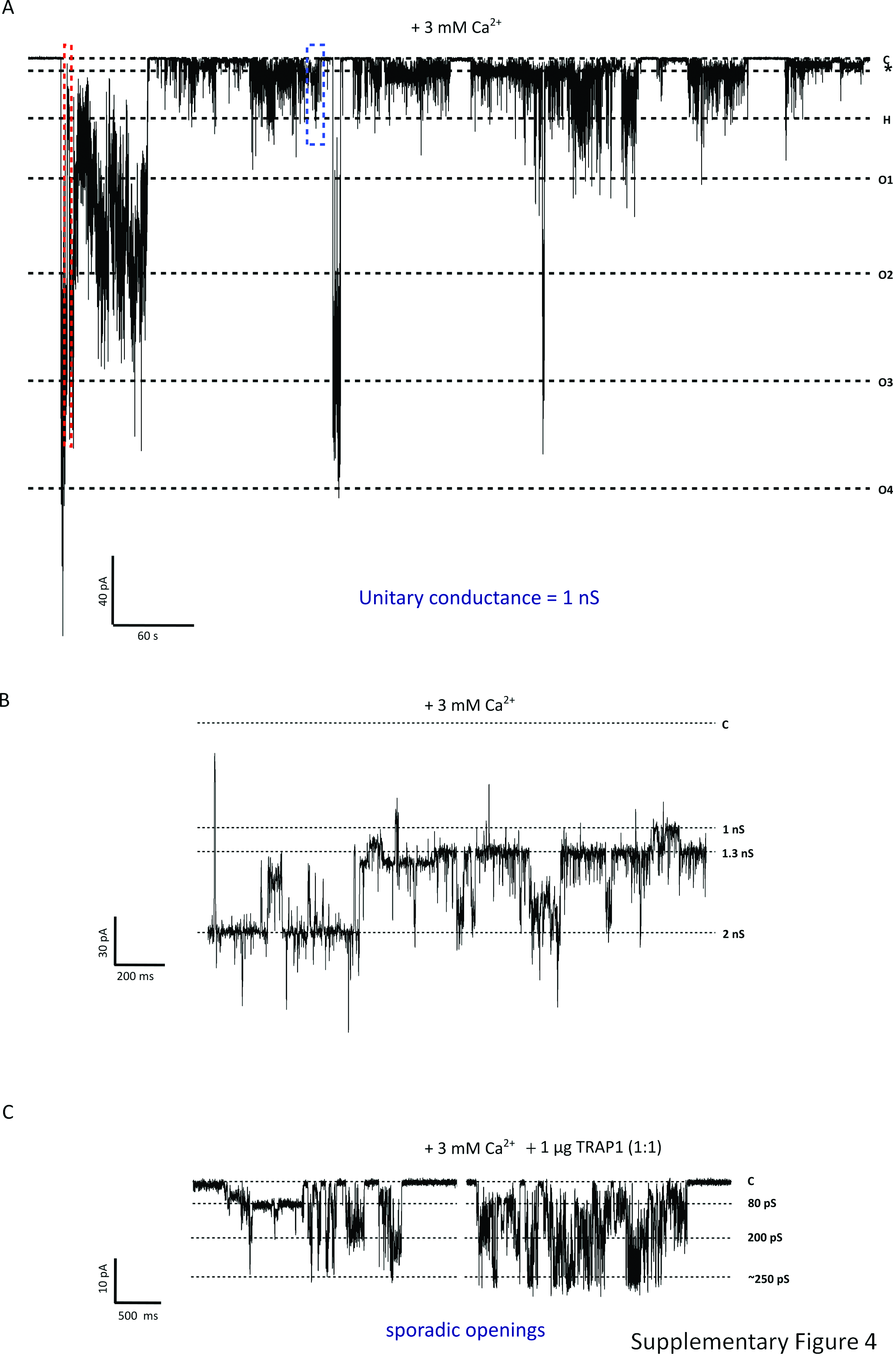

Supplement: Supplementary file 5 — Supplementary Fig. 4 [file 41418_2022_1020_MOESM5_ESM.tif]

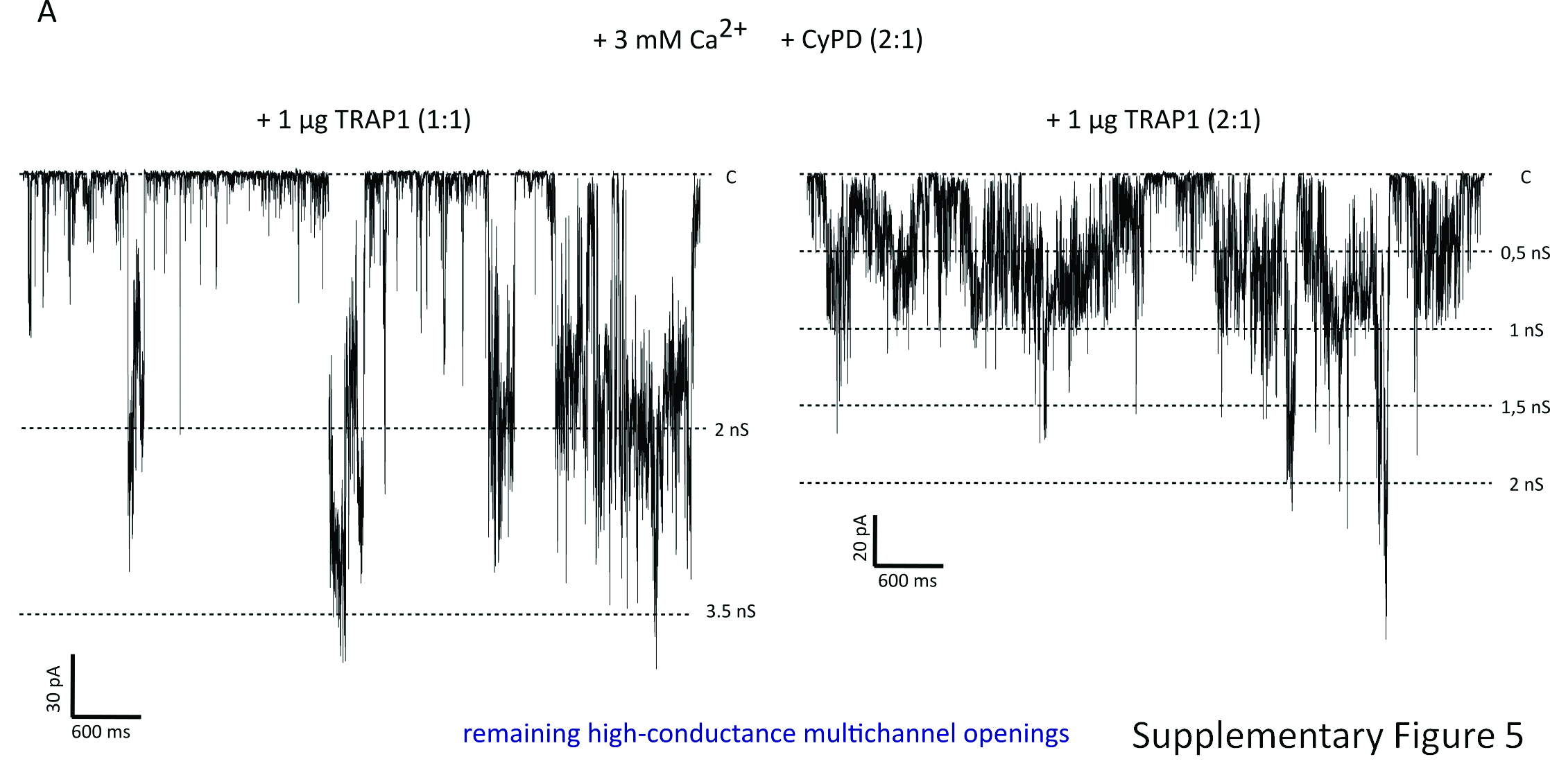

Supplement: Supplementary file 6 — Supplementary Fig. 5 [file 41418_2022_1020_MOESM6_ESM.tif]
