# Supplementary material for: The mitochondrial chaperone TRAP1 regulates F-ATP synthase channel formation
Source: Cell Death Differ. 2022 May 25;29(12):2335–46. doi: 10.1038/s41418-022-01020-0 (PMC9751095; doi:10.1038/s41418-022-01020-0)
Supplement: Supplementary file 7 — Cannino et al. marked-up [file 41418_2022_1020_MOESM7_ESM.rtf]

The mitochondrial chaperone TRAP1 regulates F-ATP synthase channel formation

Giuseppe Cannino1, Andrea Urbani1, Marco Gaspari2, Mariaconcetta Varano2, Alessandro Negro1, Antonio Filippi3, Francesco Ciscato1, Ionica Masgras1,4, Christoph Gerle5,6, Elena Tibaldi7, Anna Maria Brunati7, Giorgio Colombo8,9, Giovanna Lippe3, Paolo Bernardi1,4, Andrea Rasola1

1Department of Biomedical Sciences, University of Padova, via U. Bassi 58/B, 35131, Padova, Italy
2Research Centre for Advanced Biochemistry and Molecular Biology, Department of Experimental and Clinical Medicine, Magna Graecia University of Catanzaro, viale Europa, 88100, Catanzaro, Italy
3Department of Medicine, University of Udine, via Colugna 50, 33100, Udine, Italy
4Institute of Neuroscience, National Research Council, Viale G. Colombo 3, 35131, Padova, Italy
5Institute for Protein Research, Osaka University, 3-2 Yamadaoka, Suita, Osaka, Japan
6RIKEN SPring-8 Center, 1-1-1 Kouto, Sayo-cho, Sayo-gun, Hyogo 679-5148, Japan 
7Department of Molecular Medicine, University of Padova, via Gabelli 63, 35121, Padova, Italy
8Department of Chemistry, University of Pavia, via Taramelli 12, 27100 Pavia, Italy
9Institute of Chemical and Technological Sciences “Giulio Natta”– SCITEC, Via Mario Bianco 9, 20131 Milano, Italy

Correspondence should be addressed to:
Andrea Rasola (andrea.rasola@unipd.it)


Abstract

Binding of the mitochondrial chaperone TRAP1 to client proteins shapes bioenergetic and proteostatic adaptations of cells, but the panel of TRAP1 clients is only partially defined. Here we show that TRAP1 interacts with F-ATP synthase, the protein complex that provides most cellular ATP. TRAP1 competes with the peptidyl-prolyl cis-trans isomerase cyclophilin D (CyPD) for binding to the oligomycin sensitivity-conferring protein (OSCP) subunit of F-ATP synthase, increasing its catalytic activity and counteracting the inhibitory effect of CyPD. Electrophysiological measurements indicate that TRAP1 directly inhibits a channel activity of purified F-ATP synthase endowed with the features of the permeability transition pore (PTP) and that it reverses PTP induction by CyPD, antagonizing PTP-dependent mitochondrial depolarization and cell death. Conversely, CyPD outcompetes the TRAP1 inhibitory effect on the channel.
Our data identify TRAP1 as an F-ATP synthase regulator that can influence cell bioenergetics and survival and can be targeted in pathological conditions where these processes are dysregulated, such as cancer.


Introduction

The mitochondrial paralog of the HSP90 chaperone family TRAP1 is an important bioenergetic regulator [1] that down-modulates oxidative phosphorylation (OXPHOS) by inhibiting cytochrome c oxidase and succinate dehydrogenase (SDH) [2, 3]. A recent survey [4] identified other putative TRAP1 interactors, called clients, suggesting that its effects on cell bioenergetics are shaped by the engagement of different client subsets. TRAP1-induced formation of different protein assemblies [4, 5], together with a fine pitching of its interactions by post-translational modifications (PTMs) [3, 6, 7, 8, 9], could flexibly tune client functions, matching the activity of diverse biochemical circuits with the metabolic needs of the cell.
TRAP1 regulates the metabolic adaptations of tumor cells to the changing conditions experienced in the microenvironment. For instance, SDH inhibition by TRAP1 establishes a pseudohypoxic phenotype mediated by succinate-dependent induction of the HIF1 transcriptional program [2]. This favors neoplastic growth, and genetic or pharmacological TRAP1 inhibition hampers tumorigenicity in several models of neoplastic progression [10, 11, 12], whereas TRAP1 induction correlates with drug resistance, progression and metastatic spreading in a number of malignancies [13]. In turn, HIF1 activation increases TRAP1 expression, and TRAP1 is crucial in maintaining a low oxygen consumption rate under hypoxia, a feature whose importance extends beyond conditions of tumor growth [14]. 
In this scenario, the identification of further TRAP1 interactors is mandatory to better dissect its functions. It was recently suggested that TRAP1 clients include subunits of F-ATP synthase [4], even if a characterization of such interactions and of their functional effects was lacking. F-ATP synthase is the rotary nanomachine that synthesizes most cellular ATP by utilizing the H+ gradient created by H+ pumping coupled to electron transport across the inner membrane [15, 16, 17]. Moreover, several pieces of evidence indicate that F-ATP synthase can form a mitochondrial channel, the permeability transition pore (PTP) [18, 19, 20]. Indeed, the electrophysiological and pharmacological features of a channel formed by highly purified F-ATP synthase [20] are superimposable to those of the mitochondrial megachannel previously identified as the PTP [21], and ablation of specific F-ATP synthase subunits affects its conductance [22]. Prolonged PTP openings prompt mitochondrial depolarization and commit cells to death following a variety of stress stimuli [23, 24]. Therefore, the F-ATP synthase is at the crossroad of bioenergetic fitness and survival of cells.
TRAP1 was proposed to exert an inhibitory role on PTP opening [25, 26, 27], but the mechanisms remained elusive as the molecular nature of the channel was not defined. A proteinaceous regulator of F-ATP synthase is the mitochondrial peptidyl-prolyl cis-trans isomerase cyclophilin D (CyPD), which also interacts with TRAP1 [26, 27]. CyPD binding to the F-ATP synthase subunit OSCP results in the partial inhibition of F-ATP synthase enzymatic activity [28] and in the sensitization to PTP opening [29]. 
Here, we find that TRAP1 interaction with F-ATP synthase increases its enzymatic activity while inhibiting PTP channel opening. Our data support a model in which TRAP1 and CyPD influence bioenergetic features and survival to noxious conditions of cells by mutually outcompeting their binding to the OSCP subunit of F-ATP synthase, with important implications in pathophysiological conditions such as neoplastic transformation or adaptations to hypoxia. 


Materials and methods 

Cell and sample preparation

Experiments were performed on human U87 glioblastoma cells (ATCC) and mouse malignant peripheral nerve sheath tumor cells (sMPNST cells, kindly provided by Lu Q. Le, UTSW, Dallas, TX). TRAP1 and CyPD expression were knocked out using the lenti CRISPRv2 system [30]. Sequences for the guides used to knock out TRAP1 in U87 cells were previously reported [6]. Sequences for the guides used to knock out CyPD in sMPNST cells are: guide 1 5´ CACCGGCGACCCGTACCTGCAGCGA3´ and 5´AAACTCGCTGCAGGTACGGGTCGCC; guide 2 5´CACCGGTACACGAGCGGGTTCCCGG 3´ and 5´ AAACCCGGGAACCCGCTCGTGTACC 3´; and guide 3 5´ CACCGCCCACGTCCAAGTACACGAG 3´ and 5´ AAACCTCGTGTACTTGGACGTGGGC 3´. All guides were generated by using the CRISPR design tool (https://chopchop.cbu.uib.no/results/1571645627074.5044/). Oligonucleotide pairs were annealed and cloned into the transfer plasmid lentiCRISPRv2 (Addgene, https://www.addgene.org/52961/) and co- transfected with the three packaging plasmids pMDLg/pRRE, pRSV-Rev and pMD2.G into HEK 293T cells for viral production. Recombinant virus was collected and used to infect cells by standard methods. Infected cells were selected with 1ìg/ml puromycin. U87 cells were grown in Minimum essential medium Eagle (MEM) supplemented with 10% fetal bovine serum, glycine (4mM), pyruvate (1 mM) and non-essential amino acids. sMPNST cells were grown in Dulbecco's modified Eagle's medium (DMEM) supplemented with 10% fetal bovine serum. Cytoplasmic fractions were obtained by cells kept on ice for 20min in a lysis buffer containing 150 mM NaCl, 20 mM Tris, 5 mM EDTA‐Tris, pH 7.4 with the addition of 1% Triton X‐100, 10% glycerol and protease /phosphatase inhibitor cocktail (Merck, Darmstadt, Germany) and cleared by centrifugation at 18.000 g for 20 min, 4°C. Mitochondria were isolated after cell disruption with a glass-Teflon potter in a buffer composed of 250 mM sucrose, 10 mM Tris-HCl, 0.1 mM EGTA‐Tris, pH 7.4. 

Immunoprecipitation

Mitochondria were solubilized on ice for 30 min in lysis buffer and cleared by centrifugation at 18.000 g for 20 min, 4 °C. The supernatant was incubated overnight at 4 °C with beads (Invitrogen) previously conjugated with 1 µg of mouse monoclonal anti-human TRAP1 (sc-73604) or mouse monoclonal anti ATP50/OSCP (ab110276) antibodies. Complexes bound to beads were digested according to Bernaudo et al. [31]. Briefly, on-beads digestion was achieved by adding 200 ng trypsin in phosphate buffer (10 min at 37 °C). Alternatively, proteins were eluted with sample buffer containing 2% SDS, 50 mM Tris pH 6.8, 10 % glycerol and 0,00004% Bromophenol blue (5 min at 90 °C) and subjected to Western blotting.

LC-MS/MS

Partially digested samples were evaporated in speed vacuum and resuspended in 50 µL of HPLC water, in-solution digested and purified by C18 StageTips as described [31]. Lyophilized digests were resuspended in 29 µL of 100 mM TEAB buffer and labelled with tandem mass tags (TMT 10-plex) reagents as described below. A reference sample was created by pooling 4 µL aliquots of each sample. TMT reagents were resuspended in 100 µL of anhydrous acetonitrile; a 10 µL aliquot of each tag solution was employed to label 25 µL of each peptide mixture (one reference pool, four replicates of control samples, four replicates of TRAP1 samples). Labelled peptides were combined, fractionated by SCX StageTips and analyzed by nanoscale liquid chromatography coupled to tandem mass spectrometry as described [31]. Database search was also performed as described in the cited reference, except for the following modification: 18O was not indicated as variable modification, whereas TMT 10-plex labelling was set as fixed modification for lysine and N-terminal amino groups. Protein relative quantification was also performed in Proteome Discoverer by relying on TMT mass reporter ions. Protein hits with a minimum of two peptides identified with 95% confidence and an Xcorr of 2 were retained. Protein ratios were calculated relative to the reference sample and normalized on the basis of the content of the bait protein. Thus, the normalization factor for all protein quantification values in a particular sample was Tx/Tm, where Tx was the relative quantification value of TRAP1 for sample x and Tm was the mean relative quantification value of TRAP1 in the four sample replicates. Protein quantification and assessment of significance was determined in Prism 6.0 as described [32].

Gel filtration chromatography

Cells were disrupted on ice by sonication (2 cycles of 5 sec at 22 Hz with intervals of 15 seconds) in isotonic buffer (50 mM Tris/HCl, pH 7.5, 0.25 M sucrose, 1 mM EDTA and protease and phosphatase inhibitor cocktails). The homogenate was centrifuged for 5 min at 900 g and the precipitate (nuclei and unbroken cells) was discarded. The supernatant was centrifuged at 10000 g for 20 min. The pellet was resuspended in homogenization buffer and overlaid on cushions of 50 mM Tris/HCl, pH 7.5, containing 1 mM magnesium acetate and 1.25 M sucrose, and centrifuged at 90000 g for 60 min using a rotor of the swinging-bucket type. The Golgi apparatus fraction was collected at the 0.25 M/1.25 M sucrose-homogenate interface, while the mitochondria fraction was recovered in the 1.25 M sucrose phase. Mitochondrial fraction was lysed in buffer containing digitonin 0.7%, 20 mM Tris-HCl, pH 7.5, 2 mM EGTA, 150 mM NaCl, protease and phosphatase inhibitor cocktail, for 1 hour at 4 °C and subsequently centrifuged at 15000 g for 10 min at 4 °C. Protein concentration was determined by Bradford method. The mitochondria soluble fraction (500 µg) was applied to a FPLC-Superdex S200 (30 × 1.5 cm) equilibrated with 20 mM Tris-HCl (pH 7.5), 10% glycerol, 5 mM NaCl, 10 mM â-mercaptoethanol, protease inhibitor cocktail and supplemented with 0.5 M NaCl. The eluted fractions were analyzed by Western blot using anti-ATP50/OSCP, anti-human TRAP1, and mouse monoclonal anti-Cyclophilin (ab110324) antibodies.

Overlay assay

Purified F-ATP synthase, CyPD or TRAP1 were used. F-ATP synthase was purified from bovine heart as previously described [20]. Lauryl-maltose-neopentyl glycol (LMNG) stabilized F-ATP synthase complexes were eluted by a linear concentration gradient of 0–240 mM KCl in 40 mM HEPES pH 7.8, 150 mM sucrose, 2 mM MgCl2, 0.1 mM EDTA, 0.1 mM DTT, an d 0.05% (wt/vol) LMNG. F-ATP synthase fractions containing high amounts of native phospholipids were flash-frozen in aliquots of about 500 ìl for later use, both in overlay assays and in electrophysiology experiments.The human CypD expression system was constructed by building the pSUMO-Cyclophilin D plasmid. The nucleotide sequence encoding for human CyPD was obtained from a Human cDNA placenta library (Clontech) by PCR. The resulting vector was used to transform E. coli strain BL21(DE3)pLysS and the correctness of the cDNA was confirmed by sequencing. Human TRAP1 was produced and purified as previously described [11]. Proteins were separated by SDS-PAGE and transferred onto nitrocellulose paper. The membrane was washed briefly with distillated water and incubated in overlay blocking buffer containing 50mM Tris-HCl pH 7.5, 200mM NaCl, 3% BSA and 0,1% polyethylene glycol for 1 h at room temperature. The membrane was then incubated with overlay buffer with or without 50 µg of purified TRAP1 or CyPD for 30 min. After 4 washing for 5 min with overlay buffer the membrane was incubated with or without TRAP1/CyPD antibody in the same buffer and bound TRAP1 or CyPD were detected by Western blot analyses. 

Blue native gel electrophoresis (BN-PAGE)

Experiments were carried out on isolated mitochondria to retrieve electron transport chain (ETC) complexes. Briefly, 500 µg of mitochondria were solubilized in 50 µl of extraction buffer containing 50 mM NaCl, 2 mM 6-aminocaproic acid, 1 mM EDTA and 50 mM imidazole/HCl pH 7.0, with different concentration of digitonin and immediately centrifuged at 100000 g for 25 min at 4 °C. Supernatants were supplemented with Coomassie Blue G-250 (Serva) and applied to 1D 3-12% polyacrylamide gradient BNE (Invitrogen). After migration, the protein complexes were transferred on membrane and probed respectively with mouse monoclonal anti-ATPB (ab14730), anti SDHA (sc-166947) or anti-TRAP1 antibodies. In selected experiments, gels were stained with Coomassie Blue and monomer, dimer or oligomers of ATP-Synthase were cut and run on SDS-PAGE for protein identification by Western immunoblot or stained in-gel ATPase activity staining as reported [33] .

Measurement of kinetic constant with surface plasmon resonance (SPR)

Recombinant mature human OSCP and CyPD were produced and purified in E. coli as described [11]. SPR measurements were performed with Biacore T100 biosensor system (GE Heathcare Bioscience) using a CM5 sensor chip with immobilized CyPD. CypD was immobilized on a CM5 utilising standard amine-coupling chemistry. The amount of CypD immobilized on the activated surface was typically between 800 and 1200 response units (RU). OSCP was injected at increasing concentrations. Unspecific binding and buffer interactions were subtracted from each sensorgram and the resulting curve were fitted using a Langmiur interaction model (ProteON Manager software, Bio-rad Laboratories, Hercules, CA) to obtain binding constant.

Split GFP assay

sMPNST TRAP1 knock-out cells were grown on microscope slides and co-transfected with a combination of plasmids pcDNA3 mito-GFP1-9, pcDNA3-TRAP1-GFP10 and pcDNA3CyPD-GFP11, or pcDNA3ATP50-GFP11. Plasmids pcDNA3 mito-GFP1-9, pcDNA3-TRAP1-GFP10 and pcDNA3ATP50-GFP10 were previously described [11]; pcDNA3CyPD-GFP11 and pcDNA3ATP50-GFP11 plasmids encoding for human CyPD and OSCP respectively, were modified with the eleventh b strand of sfGFP at the C terminus (EFSGSGGGSGGGSTSEKRDHMVLLEYVTAAGITDAS). Cells were stained with 50 nm MitoTracker red for 30 min, 48 h after transfection, to detect mitochondrial network, fixed with 4% PFA and visualized with a LSM 700 confocal microscope (Zeiss, Oberkochen, Germany). For evaluating the interaction between CyPD and OSCP, sMPNST cells were transfected with the pcDNA3 mito-GFP1-9, pcDNA3CyPD-GFP11 and pcDNA3ATP50-GFP10 plasmids, stained, fixed, visualized with a fluorescence Leica DMI600B microscope (20X objective) and analyzed using LAS AF (Leica Microsystems, Wetzlar, Germany) and ImageJ® software (National Institutes of Health, University of Wisconsin, WI). The percentage of cells showing the interaction by reconstitution of GFP was calculated as the ratio between green (GFP cells) and red (total number) cells; at least 2500 cells were analyzed for each condition.

Nonyl Acridine Orange (NAO) staining

Cells, were seeded at a density of 5 x 10^4^ cells/well in a 12-wells tissue culture plate. After 24 h cells were stained for 30 min at 37 °C with NAO dye (200 nM, Merck), washed, detached with trypsin, centrifuged at 1000 g for 5 min, and suspended in the saline buffer. NAO staining was assessed by flow cytometry using the FACS Canto II flow cytometer (Becton Dickingson).

ATP hydrolysis

ATPase activity was measured monitoring the rate of NADH oxidation in 4 x 10^6^ permeabilized cells or in 50 ìg of pig heart MgATP-submitochondrial particles (SMP) in ATP regenerating buffer [28]. The buffer for cell samples contained 50 mM KCl, 50 mM Tris-HCl, 30 mM sucrose, 4 mM MgCl2, 2 mM EGTA adjusted to pH 7.4, 4 units/ml pyruvate kinase, 3 units/ml lactate dehydrogenase, 4 mM phosphoenolpyruvate, 2 mM ATP, 0.2 mM NADH. When cells were analyzed, 10 ìM alamethicin and 10 ìM sodium decavanadate were added. NADH absorbance was measured spectrophotometrically at 340 nm, 37°C. Inhibition of Mg2+‐ATPase activity was obtained by adding 1 ìM oligomycin. Values were normalized for mg of proteins. 

Calcein staining

Cells were seeded and cultured for 2 days in coverslips (105/sample) with normal culture media. The coverslips were then transferred into an open chamber and incubated in HBSS without phenol red supplemented with 8 mM CoCl2 and 0,8 ìM CsH for 10 minutes and for further 10 minutes with 0.5 ìM Calcein-AM with or without 2 ìM CsA. After calcein-AM incubation, cells were washed with PBS, incubated with HBSS in the presence of CsH and analyzed with a fluorescence microscope (Leica DMI600B). After 1 minute, the Ca2+ ionophore Calcimycin A23187 (Sigma) was added; 1 picture was recorded every 30 sec for 25 min.

Evaluation of mitochondrial membrane potential by microscopy

Mitochondrial membrane potential was tested using the fluorescent potentiometric compound tetramethylrhodamine methyl ester (TMRM, 20 nM; Invitrogen). Cells were incubated in DMEM without phenol red with 0.1% FBS and CsH (1 µM) to inhibit P-glycoproteins. Recordings were performed with a fluorescence microscope (Leica DMI600B) and analyzed using LAS AF software (Leica) as described [34]. 

Electrophysiology

Electrophysiological properties of F-ATP synthase were assessed by single-channel recording following protein insertion into artificial planar lipid bilayers by direct addition of 8 ìg of purified protein. Membranes were prepared by painting a solution of soybean asolectin (10 mg/ml in decane, Sigma) across a 250 ìm-diameter hole on a Teflon partition separating two compartments filled with a recording solution (150 mM KCl, 10 mM HEPES, pH 7.4) before membrane painting. The two compartments are defined as cis and trans, and all voltages refer to the cis side, zero being the trans (grounded) one. Currents were considered as positive when carried by cations flowing from the cis to the trans compartment. Membrane capacity ranged from 50 to 150 pF (average 100 pF) and no current leakage was detectable. F-ATP synthase was directly added to the recording chamber followed by the additions specified in the text; TRAP1 and CyPD were directly added in the recording chamber in the molar ratios to F-ATP synthase specified in the text. Data were acquired at 10 kHz through a Bilayer Clamp BC-525C amplifier (Warner Instruments, Harvard Bioscience, Inc.) and low-pass filtered at 500 Hz. Data were digitized with a Digidata 1322 A interface and pClamp software suite (all from Molecular Devices) and analyzed offline using the same software. Conductance values (G) were calculated from stable current signals corresponding to open states on the basis of Ohm's law. Maximal conductance (Gmax) was calculated from the maximal stable current level (i.e., events lasting at least 10 ms) in the recording interval.
Antibodies

Anti TRAP1, anti SDHA and anti â-actin mouse monoclonal antibodies and anti TOM20 rabbit polyclonal antibody were all from Santa Cruz (cat. #: sc-73604, sc-166947, sc-47778, sc-17764, respectively); mouse monoclonal OXPHOS antibody cocktail (cat. #: ab110411) recognizing CI (NDUFB8), CIV (COXII) and CV (á), anti-â (cat. #: ab14730), anti-b (cat. #: ab117991, abcam), and anti-OSCP (cat. #: ab110276) mouse monoclonal antibodies, and anti citrate synthase rabbit polyclonal antibody (cat. #:, ab96600) were from Abcam; rabbit polyclonal antibody anti-g (cat. #: PA5-29975) was from ThermoFisher; anti CyPD mouse monoclonal antibody was from Calbiochem (cat. #: AP1035); rabbit monoclonal antibody anti-GAPDH (cat. #: 2118) was from Cell Signaling.

Statistical analysis

Data were analyzed and presented as mean ± standard error of the mean (SEM) in all figures. Pairs of data groups were analyzed using paired and unpaired two-tailed Student's t tests. Statistical significance was determined using Origin® 8 (OriginLab, Northampton, MA). Protein quantification and assessment of significance for the mass identification was determined using Prism 6.0 with Benjamini-Hochberg procedure correction. Results with a p value lower than 0.05 were considered significant; ***p < 0.001, **p < 0.01, *p < 0.05 compared to controls. Each experiment was repeated at least three times.


Results 

TRAP1 interacts with F-ATP synthase and CyPD

We performed an unbiased nanoLC-MS/MS mass spectrometry analysis to search for TRAP1 interactors in co-immunoprecipitation experiments on human U87 glioblastoma cells, where TRAP1 displays a pro-neoplastic activity [6]. We found 95 potential TRAP1 mitochondrial interactors, including seven F-ATP synthase subunits (F1 sector subunits á, â and g, peripheral stalk subunits OSCP, d and b, and membrane subunit g) [19, 35] as well as the F-ATP synthase regulator CyPD (Fig. 1A, B; Supplementary Fig. 1, 2; Supplementary Table 1, 2). Overlay assays confirmed the binding of TRAP1 both to the a, b, g, b, OSCP and g subunits (Fig. 1C) of a highly purified F-ATP synthase preparation from bovine heart [36], and to CyPD (Fig. 1D). In a mirror experiment, purified TRAP1 [10] was found to bind CyPD (Fig. 1E), in accord with previous data [26, 27]. A direct interaction between TRAP1 and CyPD was also explored with surface plasmon resonance (SPR) (Fig. 1F). CyPD interacts with F-ATP synthase subunit OSCP [28, 37, 38, 39], which is placed on top of the “crown” region of the holoenzyme and connects the peripheral stalk with the catalytic portion of F1 [19]. SPR experiments confirmed CyPD binding to OSCP (Supplementary Fig. 2A) and revealed a direct interaction between TRAP1 and OSCP (Fig. 1G). Gel filtration chromatography analyses on U87 mitochondria showed co-localization between TRAP1 and F-ATP synthase subunits in high molecular weight fractions (Fig. 2A). Moreover, TRAP1 and CyPD associated with F-ATP synthase both in its monomeric and dimeric form (Fig. 2B), the latter constituting the physiological unit of the holoenzyme [35]. 
We then addressed whether these protein-protein interactions occur in situ by using a split-GFP approach [11, 40]. We co-expressed (1) a GFP portion (GFP1–9) fused with a mitochondrial import sequence; (2) a GFP fragment (GFP10) fused to one putative interactor; and (3) the final GFP moiety, GFP11, associated to the second potential interactor. GFP reconstitution indicates a direct interaction of the three GFP portions and therefore of the two proteins of interest, and colocalization with a matrix marker their mitochondrial localization. We carried out these tripartite split-GFP experiments on malignant peripheral nerve sheath tumor (sMPNST) cells, where TRAP1 is pro-tumorigenic [10, 11]. In order to assess for signal specificity, we first performed experiments on cells co-expressing both known interacting mitochondrial proteins as positive controls (two SDH subunits, SDHA-GFP10 and SDHB-GFP11; the green signal observed indicates a direct interaction), and non-interacting mitochondrial proteins as negative controls (OSCP-GFP10 and SDHB-GFP11; Supplementary Fig. 2B). A green staining can be observed also after co-expression of Mito-GFP1-9, CyPD-GFP10 and OSCP-GFP11, revealing a binding between CyPD and OSCP in mitochondria that is further confirmed by the merging of the green signal with the red staining of the mitochondrial probe MitoTracker Red (Supplementary Fig. 2C). The same approach was used in cells co-expressing Mito-GFP1-9 with either TRAP1-GFP10 and OSCP-GFP11 or TRAP1-GFP10 and CyPD-GFP11, revealing a direct mitochondrial interaction between OSCP and TRAP1 (Fig. 2C) and between CyPD and TRAP1 (Fig. 2D), respectively.
Altogether, these data indicate that TRAP1 interacts with F-ATP synthase and CyPD in mitochondria and suggest the presence of multimeric platforms that could confer regulatory flexibility to F-ATP synthase activity. 

TRAP1 affects the binding of CyPD to OSCP

The CyPD/OSCP interaction mildly inhibits F-ATP synthase enzymatic activity and sensitizes the PTP to opening [41, 42]. We therefore evaluated whether TRAP1 interactions with OSCP and CyPD affect F-ATP synthase functions. Gel filtration chromatography experiments detected CyPD, OSCP and TRAP1 in the same high molecular weight fractions (Fig. 3A). In TRAP1 knock-out cells all CyPD co-purified with OSCP in these fractions, whereas in TRAP1-expressing cells a low molecular weight CyPD signal was also detected, corresponding to a portion of CyPD unbound to protein complexes (Fig. 3A). Moreover, the amount of CyPD that co-immunoprecipitated with OSCP (Fig. 3B) or co-migrated with F-ATP synthase dimers and monomers (Fig. 3C) was higher in the absence of TRAP1, suggesting competition for binding. In keeping with the ability of TRAP1 to disrupt the interaction of CyPD with OSCP, split GFP experiments showed that the binding between CyPD and OSCP was enhanced by knocking-out TRAP1 or by targeting it with the highly selective TRAP1 inhibitor i5 [11] (Fig. 3D, E). Treatment with i5 also decreased the binding of TRAP1 to both OSCP and CyPD (Supplementary Fig. 3A, B). Consistently, both knocking-out CyPD and treatment with cyclosporin A (CsA), which displaces CyPD from F-ATP synthase [28], increased the interaction of TRAP1 with OSCP (Fig. 3F, G) and inhibited CyPD/OSCP binding (Supplementary Fig. 3C). These data indicate that TRAP1 and CyPD compete for the binding to OSCP and suggest that TRAP1 could counteract both the inhibitory effect of CyPD on the enzymatic activity of F-ATP synthase and its promoting effect on PTP opening.

TRAP1 increases F-ATP synthase enzymatic activity and inhibits PTP opening

We then explored the functional effects of TRAP1 binding to F-ATP synthase. Both ablation of TRAP1 and treatment with i5 inhibited the hydrolytic activity of F-ATP synthase in U87 cells (Fig. 4A), and knocking-out TRAP1 markedly decreased the in-gel hydrolytic activity of F-ATP synthase dimers without affecting the dimer/monomer protein levels (Fig. 4B). Incubation with TRAP1 also increased the hydrolytic activity of F-ATP synthase in submitochondrial particles where endogenous CyPD had been removed, and this induction was blunted by treatment with i5. In keeping with previous reports, CyPD had the opposite effect (i.e. it decreased the enzymatic activity in a CsA-sensitive way) [28], and we found that TRAP1 abrogated the inhibitory effect of CyPD (Fig. 4C). These observations are consistent with a model in which TRAP1 competes with CyPD for OSCP binding, enhancing the ATPase activity of F-ATP synthase.
By using a calcein/Co2+ assay [43], we also found that TRAP1 ablation sensitizes U87 cells to PTP opening following treatment with the Ca2+ ionophore A23187 (Fig. 4D). CyPD inhibition by CsA desensitized pore opening in TRAP1 knock-out cells, while it was ineffective in their wild-type counterparts (Fig. 4D), in accord with a competition between TRAP1 and CyPD for binding to F-ATP synthase. Prolonged PTP openings cause mitochondrial membrane depolarization and eventually cell death [23]. In keeping with an inhibitory role of TRAP1 on PTP opening, we observed that its ablation accelerates mitochondrial depolarization after cell treatment with cl-HK2pep (Fig. 4E), a peptide that induces Ca2+-dependent PTP opening, mitochondrial depolarization and cell death by displacing hexokinase 2 from endoplasmic reticulum/mitochondria contact sites [44].

TRAP1 inhibits channel opening by F-ATP synthase

We directly assessed the effect of TRAP1 on PTP opening by using a highly purified F-ATP synthase preparation, which is able to generate channel activity matching the electrophysiological features of the PTP after insertion in planar lipid bilayers [20]. We observed high-conductance channels with multiple substates upon addition of F-ATP synthase and Ca2+ to the lipid bilayers. Channel behavior was mostly flickering and in lower conductance sublevels, as previously observed in the absence of additional PTP inducers, but stable channel openings reaching up to one or more PTP full conductances were also detected (Fig. 5A, G, I; Supplementary Fig. 4A, B). Addition of TRAP1 in 1:1 to 3:1 molar ratio with F-ATP synthase reduced channel activity in a concentration-dependent way. TRAP1 inhibition occurred in 8 out of 14 experiments, as it mostly abolished the higher conductance levels leading to almost total suppression of the currents or to the maintenance of low residual currents and sporadic channel openings (Fig. 5B, C, H; Supplementary Fig. 4C), whereas the inhibitory effect of TRAP1 was less evident on the low-conductance flickering behavior of the channel (Fig. 5J, K). Residual currents were still sensitive to PTP inhibitors, such as Ba2+ (Fig. 5D). Further addition of Ca2+ could only partially restore channel activity, which was still sensitive to the inhibitory effect of TRAP1 (Fig. 5E, F). 
We also studied the effect of CyPD on the currents elicited by F-ATP synthase, and assessed a possible functional interplay between CyPD and TRAP1. We found that CyPD, in a molar ratio of 2:1 with F-ATP synthase, increased Ca2+-induced currents to high-conductance levels compatible with one or more PTPs (Fig. 6A, B). The subsequent addition of TRAP1 in a molar ratio of 1:1 with F-ATP synthase reduced channel activity (Fig. 6C), even if high conductance openings could still be observed (Supplementary Fig. 5). A further addition of TRAP1 to reach a molar ratio of 2:1 with F-ATP synthase stabilized the lower conductance states, reducing the amplitude of the remaining high-conductance channel openings (Fig. 6D; Supplementary Fig. 5B). In a mirror experiment, addition of CyPD could restore Ca2+-induced, high-conductance F-ATP synthase currents that had been reduced by TRAP1 (molar ratio of 2:1 with F-ATP synthase for both CyPD and TRAP1) (Fig. 6E-G). Further addition of CyPD up to a 5:1 molar ratio with F-ATP led to flickering openings to conductance levels compatible with one or more fully open PTPs (Fig. 6H). These data indicate that TRAP1 and CyPD compete to modulate opening of the PTP channel with opposite functional effects.


Discussion 

In the current study, we document the interaction between the molecular chaperone TRAP1, the F-ATP synthase and the peptidyl-prolyl cis-trans isomerase CyPD. We demonstrate that TRAP1 enhances the enzymatic activity of F-ATP synthase and abrogates the inhibitory effect of CyPD. Moreover, we find that TRAP1 antagonizes PTP channel openings observed with purified F-ATP synthase, whereas CyPD enhances them, and that TRAP1 and CyPD outcompete each other for PTP regulation. 
The F-ATP synthase is a fully reversible holoenzyme that synthesizes most cellular ATP via a mechanism of rotary catalysis, but it can also operate in reverse mode using the energy released by ATP hydrolysis to pump protons across the membrane. Its subunits are arranged in an FO sector embedded in the inner mitochondrial membrane, through which protons generated by respiratory complexes translocate to promote rotation, which is conveyed to the catalytic F1 sector in the matrix where ATP is produced. OSCP, together with F6, d, A6L and b subunits, forms the enzyme peripheral stalk. This acts as a stator that prevents co-rotation of F1 with FO and connects the two sectors through subunits e, f, g, a, j and k, which also form the dimerization/oligomerization interface. Central stalk components of F1 (ã, ä, and e subunits) connect the membrane ring formed by c subunits to a á3â3 hexamer in the F1 head, and enzymatic activity occurs at the interfaces between catalytic â subunits and non-catalytic á subunits [19, 45, 46]. Among potential TRAP1 interactors, we and others [4] have found the F1 components a, b and g and the peripheral stalk subunit b, g, d and OSCP, suggesting the possibility of a multifaceted regulation of the enzyme functions. The interaction between TRAP1 and OSCP might be particularly important for F-ATP synthase regulation. OSCP couples the peripheral stalk to the “crown” at the head of the F1 sector, clamping it in the correct position [45] but allowing a number of conformations during catalysis; thus, OSCP is placed in a strategical position for interacting with regulatory components of F-ATP synthase [46]. Accordingly, OSCP interacts with: (i) CyPD in a CsA-sensitive way, decreasing enzyme activity and sensitizing PTP to opening [28]; (ii) the benzodiazepine Bz-423, which mimics the effects of CyPD on both enzyme activity and PTP opening [18]; (iii) the tumor suppressor p53 during F-ATP synthase assembly [47], with p53 inducing PTP opening following CyPD binding [48] and displacing CyPD/TRAP1 interaction in vitro [27]; (iv) the NAD+-dependent deacetylase SIRT3 [39], which decreases CyPD binding by de-acetylation of OSCP Lys70 [38]; and (v) estrogen receptor  in competition with CyPD, which results in cytoprotection [37]. Moreover, OSCP Cys141 allows CyPD shielding from PTP induction by oxidants [49], whereas OSCP His112 mediates PTP inhibition by protons [50]. A cryo-EM mapping of F-ATP synthase [19] suggests a “death finger” model [51] for PTP induction, whereby CyPD binding to OSCP in the presence of Ca2+ would transmit a conformational change along the peripheral stalk to the e subunit, which would retract from the c ring, pulling out the lipid plug and causing channel opening. 
Our observations that TRAP1 binds to OSCP in competition with CyPD, counteracting the inducing effects of the latter on F-ATP synthase, add an important element to this complex picture. TRAP1 could either outcompete the CyPD/OSCP interaction or sequester CyPD away from F-ATP synthase, two non-mutually-exclusive options. We propose that OSCP acts as a hub that finely tunes both the enzymatic activity and the conversion of F-ATP synthase to the PTP by interacting with different protein regulators. The choice of the OSCP binding partner(s) would result in specific biochemical outcomes, with the effect of optimizing biological outputs to changing environmental conditions. The composition of this molecular platform could be dictated by signal transduction cascades connecting F-ATP synthase with the metabolic needs of the cell or with stress signals. Both TRAP1 and CyPD can undergo many PTMs that affect their activity, with a particular relevance in tumor models. Phosphorylation downstream to oncogenic dysregulation of Ras/ERK signalling inhibits the PTP-sensitizing effects of CyPD [52] and increases the chaperone activity and the pro-neoplastic effects of TRAP1 [6]. Additional PTMs include oxidation, S-nitrosylation, S-palmitoylation, S-glutathionylation and acetylation for CyPD [29], and S-nitrosylation and acetylation for TRAP1, as well as its formation of tetramers [53]. It is easy to envision how the combination of these modifications can finely tune access to the F-ATP synthase, resulting in facilitation of catalysis or in channel formation. It is conceivable that under conditions of nutrient and oxygen shortage, which can occur during embryonic development or in the growing tumor mass, partial suppression of F-ATP synthase activity could contribute to the establishment of an advantageous aerobic glycolysis phenotype, whereby OXPHOS is minimized in favor of glycolysis and/or glutaminolysis. Notably, we observe that neither TRAP1 nor CyPD change the levels of F-ATP synthase monomers or dimers. Hence, chaperone regulation of the enzyme must be achieved through direct protein interaction(s), allowing rapid and flexible control. 
Prolonged PTP opening constitutes a point of no return in cell death induction, making its regulation by protein-protein interactions an issue of the utmost interest in the context of a variety of pathophysiological conditions [54]. Here we demonstrate that at least two proteins, CyPD and TRAP1, regulate PTP opening in electrophysiology experiments where a purified F-ATP synthase was reconstituted into lipid bilayers. It should be noted that in the case of CyPD, the best-characterized proteinaceous PTP regulator, a direct effect on the channel activity of F-ATP synthase was never shown before. We have observed that CyPD increases F-ATP synthase channel openings and current amplitude in the presence of Ca2+, in perfect keeping with its widely documented function of PTP sensitizer. Conversely, we have documented that TRAP1 reduces Ca2+-induced channel activity of F-ATP synthase. This inhibition can be either partial, lowering the PTP conductance to sub-maximal levels, or complete, fully ablating the currents. Interestingly, TRAP1 has little if any effect on the flickering, low-conductance mode of PTP activity. These observations suggest that a direct interaction between TRAP1 and F-ATP synthase could stabilize sub-conductance levels of PTP, while inhibiting the full-conductance channel. This dual effect could be related to different conformational states of F-ATP synthase that may separately account for transient and for long-lasting PTP openings [55]. Transient openings would provide mitochondria with a fast Ca2+ release channel involved in maintaining mitochondrial and cellular Ca2+ homeostasis [56, 57, 58, 59, 60, 61], while long-lasting openings would lead to persistent mitochondrial depolarization, matrix swelling, release of apoptogenic proteins and eventually cell death [62]. In further keeping with a model where TRAP1 and CyPD compete for the binding to F-ATP synthase, each protein is able to revert the effect on PTP elicited by the other. It must be stressed that inhibition of F-ATP synthase channel by TRAP1 occurs independently of its chaperone activity, which requires ATP that was not provided in our electrophysiological experiments. Interestingly, it was proposed that in the presence of Ca2+ TRAP1 may also act as a holdase keeping clients in specific conformations independently of the canonical chaperone activity [63]. Whether a similar mechanism applies to the mode of interaction with F-ATP synthase is an intriguing possibility that deserves further investigation.
Our data predict the existence of multimeric and dynamic protein platforms that switch F-ATP synthase mode of action between catalysis and channel formation, an issue that bears on cellular bioenergetics and survival. The finding that mitochondrial interactors of TRAP1 also include TCA cycle enzymes and additional OXPHOS proteins, transporters and chaperones points to exciting directions for future investigations. Given that TRAP1 is expressed in many cancers, disruption of its regulatory interactions by highly selective inhibitors [64] may help develop innovative anti-neoplastic treatments. 

Acknowledgements 

We thank Elena Trevisan for invaluable technical assistance. 

Funding statement

A.R., P.B. and G.Co. are supported by Associazione Italiana Ricerca Cancro (AIRC grants IG 2017/20749, IG 2019/23129 and IG 2017/20019, respectively) and by MIUR (Italy) Progetti di Ricerca di Rilevante Interesse Nazionale 2017LHFW42 (to P.B.). F.C. is recipient of a Young Investigator Award Grant from Children's Tumor Foundation.


Author contributions

G.Ca.: conceptualization, visualization, methodology, investigation, formal analysis, writing-original draft; A.U.: investigation, formal analysis; M.G.: methodology, formal analysis; M.V.: investigation, formal analysis; A.N.: methodology, investigation, formal analysis; A.F.: investigation, formal analysis; F.C.: investigation, formal analysis; I.M.: conceptualization, formal analysis; C.G., G.Co.: methodology, resources; E.T.: investigation, formal analysis; A.M.B.: methodology, formal analysis; G.L.: conceptualization, methodology, visualization, formal analysis; P.B.: conceptualization, review and editing original draft, funding acquisition; A.R.: conceptualization, writing original draft and subsequent review and editing of the text, funding acquisition, project administration, supervision.


Competing interests

The Authors declare no competing interests.

Ethic statement 

Our studies did not include either human participants, data or tissues, or animal studies.


Data availability

All data needed to evaluate the conclusions in the paper are present in the paper and/or the Supplementary Materials. Additional data related to this paper may be requested from the authors.

Supplementary information is available at Cell Death & Differentiation's website.


References

1.	Rasola A, Neckers L, Picard D. Mitochondrial oxidative phosphorylation TRAP(1)ped in tumor cells. Trends Cell Biol. 2014;24:455-63.
2.	Sciacovelli M, Guzzo G, Morello V, Frezza C, Zheng L, Nannini N, et al. The mitochondrial chaperone TRAP1 promotes neoplastic growth by inhibiting succinate dehydrogenase. Cell Metab. 2013;17:988-99.
3.	Yoshida S, Tsutsumi S, Muhlebach G, Sourbier C, Lee MJ, Lee S, et al. Molecular chaperone TRAP1 regulates a metabolic switch between mitochondrial respiration and aerobic glycolysis. Proc Natl Acad Sci U S A. 2013;110:E1604-12.
4.	Joshi A, Dai L, Liu Y, Lee J, Ghahhari NM, Segala G, et al. The mitochondrial HSP90 paralog TRAP1 forms an OXPHOS-regulated tetramer and is involved in mitochondrial metabolic homeostasis. BMC Biol. 2020;18:10.
5.	Liu Y, Sun M, Elnatan D, Larson A, Agard DA. Cryo-EM analysis of human mitochondrial Hsp90 in multiple tetrameric states. BioRxiv. 2020;11.04.368837.
6.	Masgras I, Ciscato F, Brunati AM, Tibaldi E, Indraccolo S, Curtarello M, et al. Absence of Neurofibromin Induces an Oncogenic Metabolic Switch via Mitochondrial ERK-Mediated Phosphorylation of the Chaperone TRAP1. Cell Rep. 2017;18:659-72.
7.	Park HK, Hong JH, Oh YT, Kim SS, Yin J, Lee AJ, et al. Interplay between TRAP1 and Sirtuin-3 Modulates Mitochondrial Respiration and Oxidative Stress to Maintain Stemness of Glioma Stem Cells. Cancer Res. 2019;79:1369-82.
8.	Pridgeon JW, Olzmann JA, Chin LS, Li L. PINK1 protects against oxidative stress by phosphorylating mitochondrial chaperone TRAP1. PLoS Biol. 2007;5:e172.
9.	Rizza S, Montagna C, Cardaci S, Maiani E, Di Giacomo G, Sanchez-Quiles V, et al. S-nitrosylation of the Mitochondrial Chaperone TRAP1 Sensitizes Hepatocellular Carcinoma Cells to Inhibitors of Succinate Dehydrogenase. Cancer Res. 2016;76:4170-82.
10.	Sanchez-Martin C, Menon D, Moroni E, Ferraro M, Masgras I, Elsey J, et al. Honokiol bis-dichloroacetate is a selective allosteric inhibitor of the mitochondrial chaperone TRAP1. Antioxid Redox Signal. 2020;34:505-16.
11.	Sanchez-Martin C, Moroni E, Ferraro M, Laquatra C, Cannino G, Masgras I, et al. Rational Design of Allosteric and Selective Inhibitors of the Molecular Chaperone TRAP1. Cell Rep. 2020;31:107531.
12.	Xie S, Wang X, Gan S, Tang X, Kang X, Zhu S. The Mitochondrial Chaperone TRAP1 as a Candidate Target of Oncotherapy. Front Oncol. 2020;10:585047.
13.	Masgras I, Sanchez-Martin C, Colombo G, Rasola A. The Chaperone TRAP1 As a Modulator of the Mitochondrial Adaptations in Cancer Cells. Front Oncol. 2017;7:58.
14.	Laquatra C, Sanchez-Martin C, Dinarello A, Cannino G, Minervini G, Moroni E, et al. HIF1alpha-dependent induction of the mitochondrial chaperone TRAP1 regulates bioenergetic adaptations to hypoxia. Cell Death Dis. 2021;12:434.
15.	Boyer PD. A research journey with ATP synthase. J Biol Chem. 2002;277:39045-61.
16.	Kuhlbrandt W. Structure and Mechanisms of F-Type ATP Synthases. Annu Rev Biochem. 2019;88:515-49.
17.	Mitchell P. Keilin's respiratory chain concept and its chemiosmotic consequences. Science. 1979;206:1148-59.
18.	Giorgio V, von Stockum S, Antoniel M, Fabbro A, Fogolari F, Forte M, et al. Dimers of mitochondrial ATP synthase form the permeability transition pore. Proc Natl Acad Sci U S A. 2013;110:5887-92.
19.	Pinke G, Zhou L, Sazanov LA. Cryo-EM structure of the entire mammalian F-type ATP synthase. Nat Struct Mol Biol. 2020;27:1077-85.
20.	Urbani A, Giorgio V, Carrer A, Franchin C, Arrigoni G, Jiko C, et al. Purified F-ATP synthase forms a Ca(2+)-dependent high-conductance channel matching the mitochondrial permeability transition pore. Nat Commun. 2019;10:4341.
21.	Szabo I, Zoratti M. Mitochondrial channels: ion fluxes and more. Physiol Rev. 2014;94:519-608.
22.	Carrer A, Tommasin L, Sileikyte J, Ciscato F, Filadi R, Urbani A, et al. Defining the molecular mechanisms of the mitochondrial permeability transition through genetic manipulation of F-ATP synthase. Nat Commun. 2021;12:4835.
23.	Bernardi P, Rasola A, Forte M, Lippe G. The Mitochondrial Permeability Transition Pore: Channel Formation by F-ATP Synthase, Integration in Signal Transduction, and Role in Pathophysiology. Physiol Rev. 2015;95:1111-55.
24.	Rasola A, Bernardi P. The mitochondrial permeability transition pore and its involvement in cell death and in disease pathogenesis. Apoptosis. 2007;12:815-33.
25.	Guzzo G, Sciacovelli M, Bernardi P, Rasola A. Inhibition of succinate dehydrogenase by the mitochondrial chaperone TRAP1 has anti-oxidant and anti-apoptotic effects on tumor cells. Oncotarget. 2014;5:11897-908.
26.	Kang BH, Plescia J, Dohi T, Rosa J, Doxsey SJ, Altieri DC. Regulation of tumor cell mitochondrial homeostasis by an organelle-specific Hsp90 chaperone network. Cell. 2007;131:257-70.
27.	Lebedev I, Nemajerova A, Foda ZH, Kornaj M, Tong M, Moll UM, et al. A Novel In Vitro CypD-Mediated p53 Aggregation Assay Suggests a Model for Mitochondrial Permeability Transition by Chaperone Systems. J Mol Biol. 2016;428:4154-67.
28.	Giorgio V, Bisetto E, Soriano ME, Dabbeni-Sala F, Basso E, Petronilli V, et al. Cyclophilin D modulates mitochondrial F0F1-ATP synthase by interacting with the lateral stalk of the complex. J Biol Chem. 2009;284:33982-8.
29.	Amanakis G, Murphy E. Cyclophilin D: An Integrator of Mitochondrial Function. Front Physiol. 2020;11:595.
30.	Sanjana NE, Shalem O, Zhang F. Improved vectors and genome-wide libraries for CRISPR screening. Nat Methods. 2014;11:783-84.
31.	Bernaudo F, Monteleone F, Mesuraca M, Krishnan S, Chiarella E, Scicchitano S, et al. Validation of a novel shotgun proteomic workflow for the discovery of protein-protein interactions: focus on ZNF521. J Proteome Res. 2015;14:1888-99.
32.	Taverna D, Mignogna C, Gabriele C, Santise G, Donato G, Cuda G, et al. An optimized procedure for on-tissue localized protein digestion and quantification using hydrogel discs and isobaric mass tags: analysis of cardiac myxoma. Anal Bioanal Chem. 2017;409:2919-30.
33.	Bisetto E, Di Pancrazio F, Simula MP, Mavelli I, Lippe G. Mammalian ATPsynthase monomer versus dimer profiled by blue native PAGE and activity stain. Electrophoresis. 2007;28:3178-85.
34.	Ciscato F, Chiara F, Filadi R, Rasola A. Analysis of the Effects of Hexokinase 2 Detachment From Mitochondria-Associated Membranes with the Highly Selective Peptide HK2pep. Bio Protoc. 2021;11:e4087.
35.	Spikes TE, Montgomery MG, Walker JE. Structure of the dimeric ATP synthase from bovine mitochondria. Proc Natl Acad Sci U S A. 2020;117:23519-26.
36.	Jiko C, Davies KM, Shinzawa-Itoh K, Tani K, Maeda S, Mills DJ, et al. Bovine F1Fo ATP synthase monomers bend the lipid bilayer in 2D membrane crystals. Elife. 2015;4:e06119.
37.	Burstein SR, Kim HJ, Fels JA, Qian L, Zhang S, Zhou P, et al. Estrogen receptor beta modulates permeability transition in brain mitochondria. Biochim Biophys Acta Bioenerg. 2018;1859:423-33.
38.	Lee CF, Chavez JD, Garcia-Menendez L, Choi Y, Roe ND, Chiao YA, et al. Normalization of NAD+ Redox Balance as a Therapy for Heart Failure. Circulation. 2016;134:883-94.
39.	Yang W, Nagasawa K, Munch C, Xu Y, Satterstrom K, Jeong S, et al. Mitochondrial Sirtuin Network Reveals Dynamic SIRT3-Dependent Deacetylation in Response to Membrane Depolarization. Cell. 2016;167:985-1000 e21.
40.	Cabantous S, Nguyen HB, Pedelacq JD, Koraichi F, Chaudhary A, Ganguly K, et al. A new protein-protein interaction sensor based on tripartite split-GFP association. Sci Rep. 2013;3:2854.
41.	Giorgio V, Fogolari F, Lippe G, Bernardi P. OSCP subunit of mitochondrial ATP synthase: role in regulation of enzyme function and of its transition to a pore. Br J Pharmacol. 2019;176:4247-57.
42.	Giorgio V, Soriano ME, Basso E, Bisetto E, Lippe G, Forte MA, et al. Cyclophilin D in mitochondrial pathophysiology. Biochim Biophys Acta. 2010;1797:1113-8.
43.	Petronilli V, Miotto G, Canton M, Brini M, Colonna R, Bernardi P, et al. Transient and long-lasting openings of the mitochondrial permeability transition pore can be monitored directly in intact cells by changes in mitochondrial calcein fluorescence. Biophys J. 1999;76:725-34.
44.	Ciscato F, Filadi R, Masgras I, Pizzi M, Marin O, Damiano N, et al. Hexokinase 2 displacement from mitochondria-associated membranes prompts Ca(2+) -dependent death of cancer cells. EMBO Rep. 2020;21:e49117.
45.	He J, Carroll J, Ding S, Fearnley IM, Montgomery MG, Walker JE. Assembly of the peripheral stalk of ATP synthase in human mitochondria. Proc Natl Acad Sci U S A. 2020;117:29602-08.
46.	Murphy BJ, Klusch N, Langer J, Mills DJ, Yildiz O, Kuhlbrandt W. Rotary substates of mitochondrial ATP synthase reveal the basis of flexible F1-Fo coupling. Science. 2019;364:eaaw9128.
47.	Bergeaud M, Mathieu L, Guillaume A, Moll UM, Mignotte B, Le Floch N, et al. Mitochondrial p53 mediates a transcription-independent regulation of cell respiration and interacts with the mitochondrial F(1)F0-ATP synthase. Cell Cycle. 2013;12:2781-93.
48.	Vaseva AV, Marchenko ND, Ji K, Tsirka SE, Holzmann S, Moll UM. p53 opens the mitochondrial permeability transition pore to trigger necrosis. Cell. 2012;149:1536-48.
49.	Carraro M, Jones K, Sartori G, Schiavone M, Antonucci S, Kucharczyk R, et al. The Unique Cysteine of F-ATP Synthase OSCP Subunit Participates in Modulation of the Permeability Transition Pore. Cell Rep. 2020;32:108095.
50.	Antoniel M, Jones K, Antonucci S, Spolaore B, Fogolari F, Petronilli V, et al. The unique histidine in OSCP subunit of F-ATP synthase mediates inhibition of the permeability transition pore by acidic pH. EMBO Rep. 2018;19:257-68.
51.	Gerle C. On the structural possibility of pore-forming mitochondrial FoF1 ATP synthase. Biochim Biophys Acta. 2016;1857:1191-96.
52.	Rasola A, Sciacovelli M, Chiara F, Pantic B, Brusilow WS, Bernardi P. Activation of mitochondrial ERK protects cancer cells from death through inhibition of the permeability transition. Proc Natl Acad Sci U S A. 2010;107:726-31.
53.	Masgras I, Laquatra C, Cannino G, Serapian SA, Colombo G, Rasola A. The molecular chaperone TRAP1 in cancer: From the basics of biology to pharmacological targeting. Semin Cancer Biol. 2021;S1044-579X(21)00196-6.
54.	Rasola A, Bernardi P. The mitochondrial permeability transition pore and its adaptive responses in tumor cells. Cell Calcium. 2014;56:437-45.
55.	Zoratti M, Szabo I. The mitochondrial permeability transition. Biochim Biophys Acta. 1995;1241:139-76.
56.	Agarwal A, Wu PH, Hughes EG, Fukaya M, Tischfield MA, Langseth AJ, et al. Transient Opening of the Mitochondrial Permeability Transition Pore Induces Microdomain Calcium Transients in Astrocyte Processes. Neuron. 2017;93:587-605 e7.
57.	Barsukova A, Komarov A, Hajnoczky G, Bernardi P, Bourdette D, Forte M. Activation of the mitochondrial permeability transition pore modulates Ca(2+) responses to physiological stimuli in adult neurons. Eur J Neurosci. 2010;33:831-42.
58.	Boyman L, Coleman AK, Zhao G, Wescott AP, Joca HC, Greiser BM, et al. Dynamics of the mitochondrial permeability transition pore: Transient and permanent opening events. Arch Biochem Biophys. 2019;666:31-39.
59.	Elrod JW, Wong R, Mishra S, Vagnozzi RJ, Sakthievel B, Goonasekera SA, et al. Cyclophilin D controls mitochondrial pore-dependent Ca(2+) exchange, metabolic flexibility, and propensity for heart failure in mice. J Clin Invest. 2010;120:3680-7.
60.	Lu X, Kwong JQ, Molkentin JD, Bers DM. Individual Cardiac Mitochondria Undergo Rare Transient Permeability Transition Pore Openings. Circ Res. 2016;118:834-41.
61.	Ying Z, Xiang G, Zheng L, Tang H, Duan L, Lin X, et al. Short-Term Mitochondrial Permeability Transition Pore Opening Modulates Histone Lysine Methylation at the Early Phase of Somatic Cell Reprogramming. Cell Metab. 2018;28:935-45 e5.
62.	Petronilli V, Penzo D, Scorrano L, Bernardi P, Di Lisa F. The mitochondrial permeability transition, release of cytochrome c and cell death. Correlation with the duration of pore openings in situ. J Biol Chem. 2001;276:12030-4.
63.	Elnatan D, Betegon M, Liu Y, Ramelot T, Kennedy MA, Agard DA. Symmetry broken and rebroken during the ATP hydrolysis cycle of the mitochondrial Hsp90 TRAP1. Elife. 2017;6:e25235.
64.	Sanchez-Martin C, Serapian SA, Colombo G, Rasola A. Dynamically Shaping Chaperones. Allosteric Modulators of HSP90 Family as Regulatory Tools of Cell Metabolism in Neoplastic Progression. Front Oncol. 2020;10:1177.


Figure legends

Fig. 1 TRAP1 interacts with F-ATP synthase and CyPD. A NanoLC-MS/MS mass spectrometry analysis of TRAP1 interactors performed on mitochondria from U87 cells identifies F-ATP synthase subunits and CyPD. B Western immunoblot analysis of mitochondrial markers in human U87 glioblastoma cells with or without TRAP1 (infected with a vector containing a scrambled sequence, SCR, or with a knock-out one, KO, respectively) shows no changes in protein content. C-E Overlay assays. In C, silver staining (left) and Western immunoblot of purified F-ATP synthase (center) were carried out on enzyme subunits separated by SDS-PAGE. Samples transferred onto nitrocellulose were then incubated with or without the purified TRAP1 protein and binding was detected with an anti-TRAP1 antibody (right). In D, Western immunoblot on purified CyPD followed by sample transfer onto nitrocellulose, incubation with or without the purified TRAP1 protein and detection with anti-TRAP1 antibody; in E, Western immunoblot on purified TRAP1 followed by sample transfer onto nitrocellulose, incubation with or without the purified CyPD protein and detection with anti-CyPD antibody. Casein was used as a negative control in all overlay assays. F, G SPR analysis of the binding between CyPD and TRAP1 (F) and between OSCP and TRAP1 (G). Representative schemes of the SPR procedure followed to measure CyPD or OSCP binding to Myc-TRAP1 are shown in the upper left part of each figure. Representative sensorgrams obtained after the injection of the reported concentrations of CyPD or OSCP over Myc-TRAP1, previously immobilized on the surface of the CM5 sensor chips, are reported in the lower left part of each figure. The average response from 0-180 s was plotted as a function of CyPD/OSCP concentration and fitted to a Langmuir interaction model to determine Ka (right part of each figure).

Fig. 2 TRAP1 interaction with OSCP and CyPD. A Gel filtration chromatography analysis of TRAP1 distribution in mitochondria of human U87 glioblastoma cells. OSCP and â subunits were used to assess the presence of F-ATP synthase. B BN-PAGE performed on isolated mitochondria (left) showing F-ATP synthase monomers and dimers (M and D, respectively). M and D bands were cut, run on SDS-PAGE and probed for the presence of F-ATP synthase subunits ã and OSCP, TRAP1 and CyPD. C, D Tripartite split GFP experiment on sMPNST cells co-transfected with GFP1–9 fused to a mitochondrial import sequence, together with GFP10-TRAP1 and GFP11-OSCP (C), or with GFP10-TRAP1 and GFP11-CyPD (D). Green dots indicate mitochondrial interaction between TRAP1 and OSCP (C) or TRAP1 and CyPD (D). MitoTracker (red dots) was used to stain mitochondria; superimposition of MitoTracker and GFP signal is shown as yellow dots and indicates that protein interaction occurs in mitochondria.

Fig. 3 TRAP1 and CyPD compete for interaction with OSCP. A Gel filtration chromatography analysis of CyPD and OSCP distribution in mitochondria of human U87 glioblastoma cells with or without TRAP1 (SCR and KO, respectively). B Immunoprecipitation of OSCP from mitochondria of U87 cells. On the right, quantification of the amount of CyPD bound to OSCP. Data are presented as mean ± SEM of 4 independent experiments; **p < 0.01 with a paired two-tailed Student's t test. C BN-PAGE and Western immunoblotting of F-ATP synthase extracted from mitochondria of U87 cells. D (dimer) and M (monomer) bands were cut, run on SDS-PAGE and probed with antibodies for TRAP1, CyPD and F-ATP synthase subunits ã and OSCP. Histograms refer to the mean ± SEM of CyPD bound to dimers (center) or monomers (right) normalized for the content of OSCP subunit of 4 independent experiments. ∗p< 0.05 with a paired two-tailed Student's t test. D, F Western immunoblots of mitochondrial markers from sMPNST cells with or without TRAP1 (D; SCR and KO, respectively), and with or without CyPD (F; SCR and KO, respectively); actin was used as a loading control. E, G Tripartite split GFP experiment on sMPNST cells co-transfected with GFP1–9 fused to a mitochondrial import sequence, together with GFP10-CyPD and GFP11-OSCP (E), or with GFP10-TRAP1 and GFP11-OSCP (G). Experiment analysis was carried out as in Figure 2C, D. Quantification of GFP-positive cells was carried out without or with treatment with 100 µM TRAP1 inhibitor i5 (histograms in E; cells analyzed >1300) or without or with treatment with 2 µM CsA (histograms in G; cells analyzed >1500). Data are presented as mean ± SEM. ∗∗p< 0.01 with a paired two-tailed Student's t test.

Fig. 4 Effect of TRAP1 interaction on F-ATP synthase enzymatic activity and PTP opening. A Oligomycin-sensitive ATPase activity on human U87 glioblastoma cells with or without TRAP1 (SCR and KO, respectively). Where indicated, the TRAP1 inhibitor i5 was used at 25 µM. The histogram reports the mean ± SEM of 4 independent experiments. B BN-PAGE followed by in gel ATPase activity (top left) on U87 mitochondria extracted with the indicated concentrations of digitonin. Quantification of ATPase activity is reported as the mean dimer/monomer ratio from three independent measurements; *p< 0.05, **p<0.01 ***p<0.005 (bottom, left). BN-PAGE followed by Western immunoblotting (top, right) was probed with antibodies for F-ATP synthase subunit â to detect enzyme monomers, dimers and oligomers; anti-SDHA antibody was used as a loading control. C ATPase activity measured in submitochondrial particles (SMPs) from pig heart pre-incubated with 1 nmol of TRAP1 or CyPD per mg of protein alone or sequentially for 5 min at 37°C. Where indicated, the TRAP1 inhibitor i5 (100 mM) or CsA (4 mM) were preincubated for 5 min at 37°C. Data are shown as percentage changes with respect to untreated SMPs. D Calcein cobalt assay of PTP opening on U87 cells. Cells were stained with calcein AM at time zero; 2 mM Ca2+ ionophore A23187 was added where indicated in the absence or in the presence of 2 mM CsA. Image frames were collected at 30-s intervals and fluorescence values (mean ± SEM n=4) were quantified. E Mitochondrial membrane potential assessment in U87 cells after the addition of 2 µM cl-HK2pep. Cells were stained with TMRM probe (20 nM + 1 µM CsH); data are reported as mean ± SD; **p<0.01 with a TwoWay Anova p<0,001, Bonferroni post-test analysis. 
Fig. 5 Effect of TRAP1 on F-ATP synthase channel activity. A-F High conductance channel activity, corresponding to one full-conductance PTP, detected after addition to the cis side of the planar lipid bilayer of 8 µg F-ATP synthase and of 3 mM Ca2+ (A; Vcis = –60 mV). The effects on channel activity of subsequent additions of TRAP1 (B: molar ratio with F-ATP synthase of 1:1; Gmax = 500 pS; C: molar ratio with F-ATP synthase of 2:1; Gmax = 450 pS), 5 mM Ba2+ (D), further 3 mM Ca2+ (E; Gmax = 250 pS); further 1 µg TRAP1 (F; molar ratio with F-ATP synthase of 3:1; Gmax = 100 pS) are reported. G, H Very high and complex channel activity, corresponding to up to three PTPs at full-conductance (unitary conductance 1 nS), detected after addition of 3 mM Ca2+ (g; Vcis = –60 mV); see Supplementary Fig. 4 for the whole trace. Current levels corresponding to a single PTP in full-conductance and half-conductance are also visible (1 nS and 600 pS), as well as levels corresponding to other multiple PTP openings (2.2 nS, 3 nS). On the right, channel activity at lower conductance levels is shown at a higher magnification. In H, addition of TRAP1 (at a molar ratio with F-ATP synthase of 1:1) led to almost total current suppression. Sporadic channel openings are reported in Supplementary Fig. 4. I-K Flickering channel activity at low conductance detected after addition of 3 mM Ca2+. In (I), the most represented conductance level was at 150 pS, while the maximal conductance state was at 350 pS. The effects on channel activity of subsequent additions of TRAP1 (J: molar ratio with F-ATP synthase of 1:1; Gmax = 250 pS; K: molar ratio with F-ATP synthase of 2:1; Gmax = 250 pS) are reported. 
All along the figure, the prevalent conductance levels are marked with dotted lines and the conductance values are reported; the closed state is denoted with C.

Fig. 6 Opposite effects and competition of TRAP1 and CyPD on F-ATP synthase channel activity. A-D High conductance, flickering channel activity detected after direct incorporation of 16 µg F-ATP synthase in the planar lipid bilayer and addition of 3 mM Ca2+ (A; Vcis = –60 mV). The effects on channel activity of subsequent additions of CyPD (B; molar ratio with F-ATP synthase of 2:1) and TRAP1 (C: molar ratio with F-ATP synthase of 1:1; D: molar ratio with F-ATP synthase of 2:1) are reported. E-H High conductance, flickering channel activity detected in the presence of 3 mM Ca2+ (E; Vcis = –60 mV). The effects on channel activity of subsequent additions of TRAP1 (F; molar ratio with F-ATP synthase of 2:1) and CyPD (G: molar ratio with F-ATP synthase of 2:1; H: molar ratio with F-ATP synthase of 5:1) are reported. 
In all figure panels, the prevalent conductance levels are marked with dotted lines and the conductance values are indicated; the closed state is denoted with C. Either TRAP1 or CyPD were added at increasing concentrations after Ca2+-induced channel activity was detected.
