# Supplementary material for: The mitochondrial chaperone TRAP1 regulates F-ATP synthase channel formation
Source: Cell Death Differ. 2022 May 25;29(12):2335–46. doi: 10.1038/s41418-022-01020-0 (PMC9751095; doi:10.1038/s41418-022-01020-0)
Supplement: Supplementary file 9 — Uncropped Western blots [file 41418_2022_1020_MOESM9_ESM.pdf]

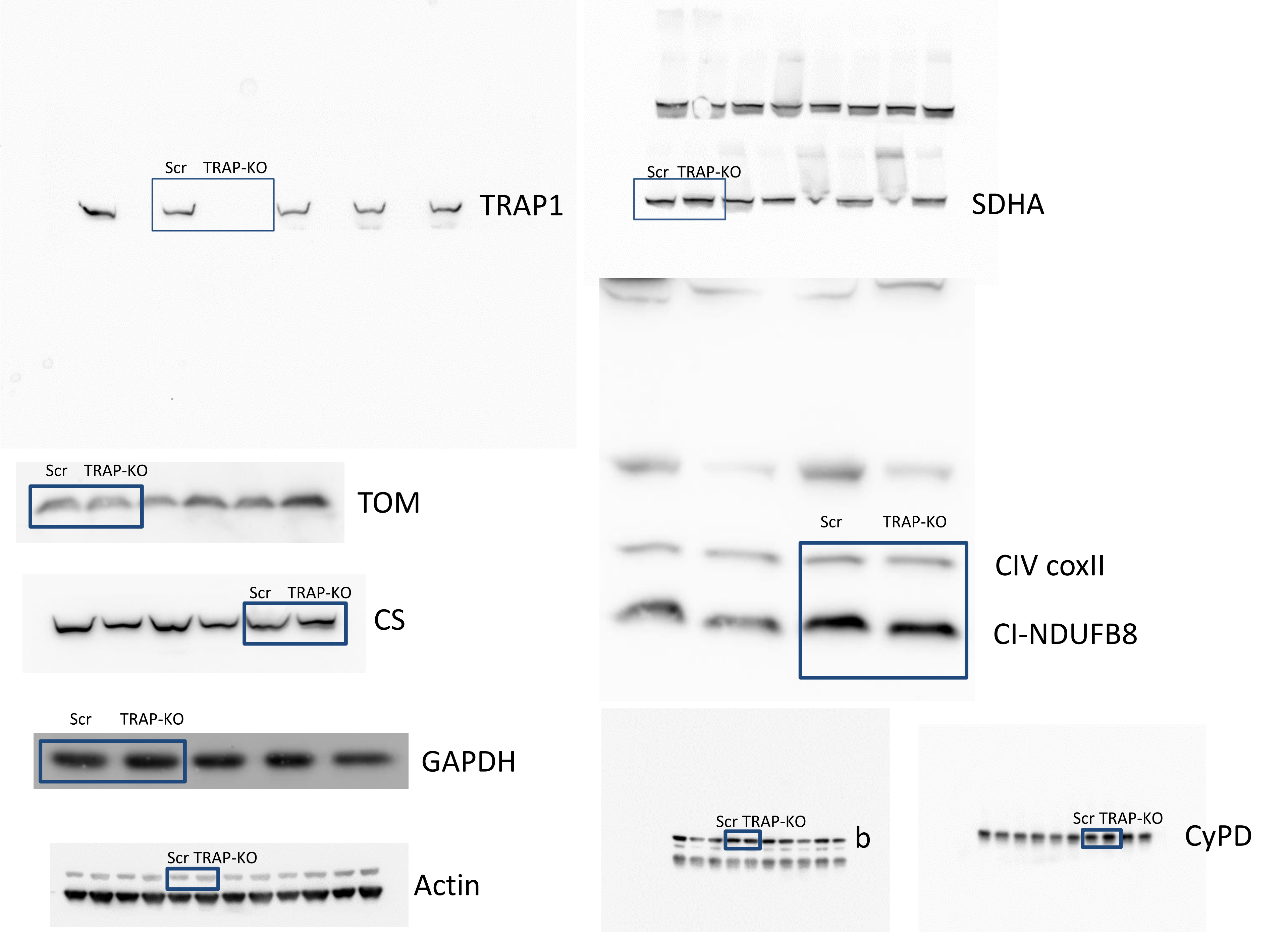
Scr TRAP-KO
TRAP1
Scr TRAP-KO
SDHA
Scr TRAP-KO
TOM
Scr TRAP-KO
CS
Scr TRAP-KO
CIV coxII
CI-NDUFB8
Scr TRAP-KO
GAPDH
Scr TRAP-KO
Actin
Scr TRAP-KO
b
Scr TRAP-KO
CyPD

Fig 1 B

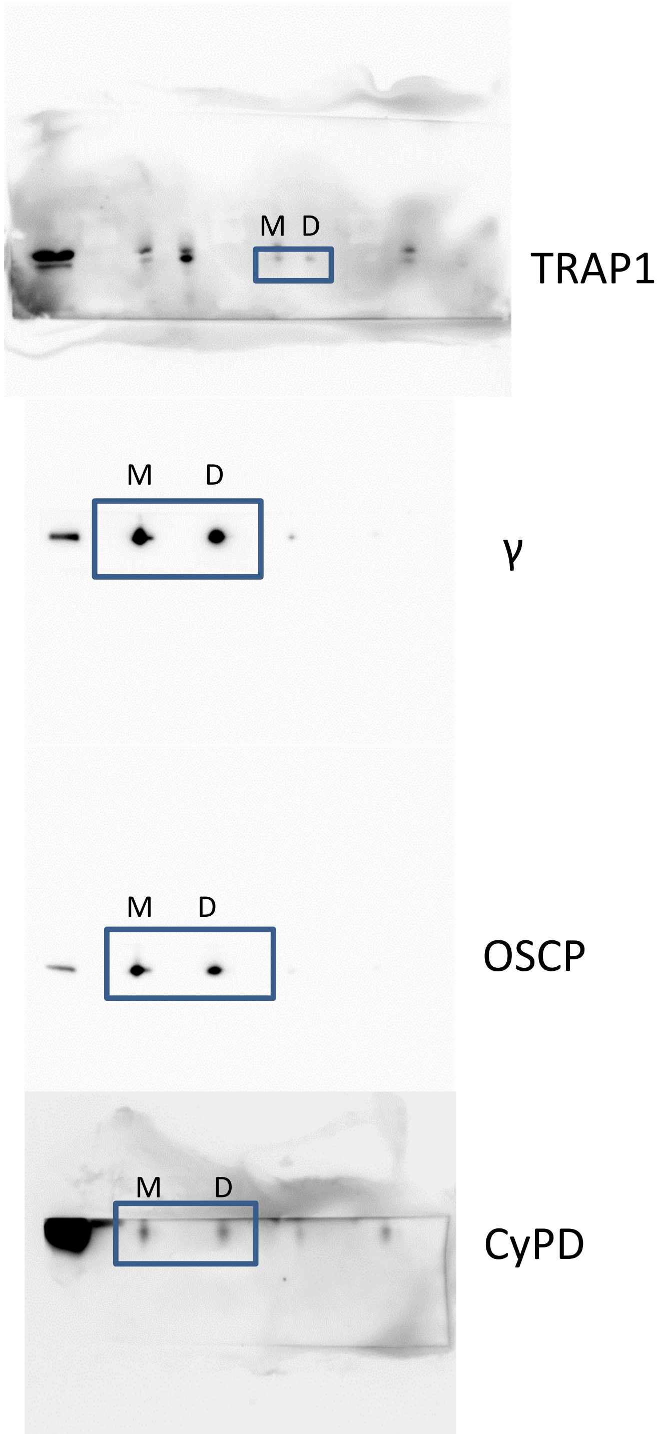

Fig 3C

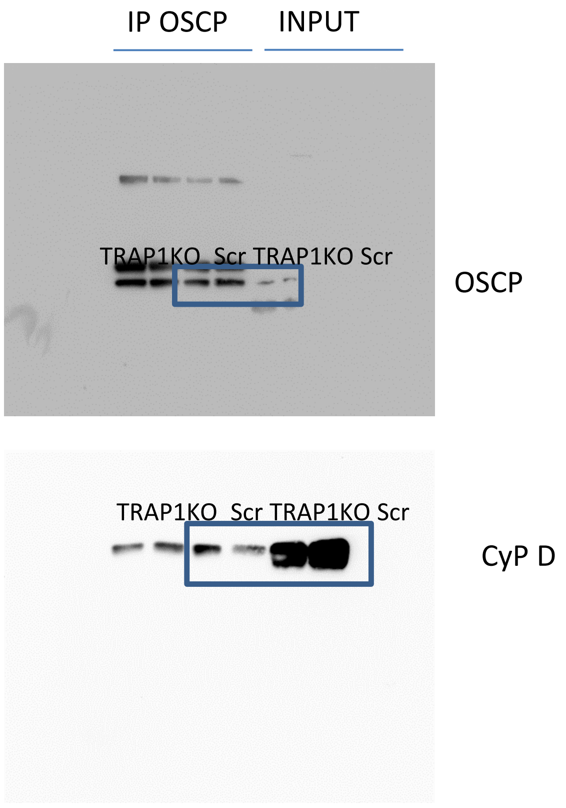
IP OSCP
INPUT
TRAP1KO Scr TRAP1KO Scr
OSCP
TRAP1KO Scr TRAP1KO Scr
CyP D

Fig 3B

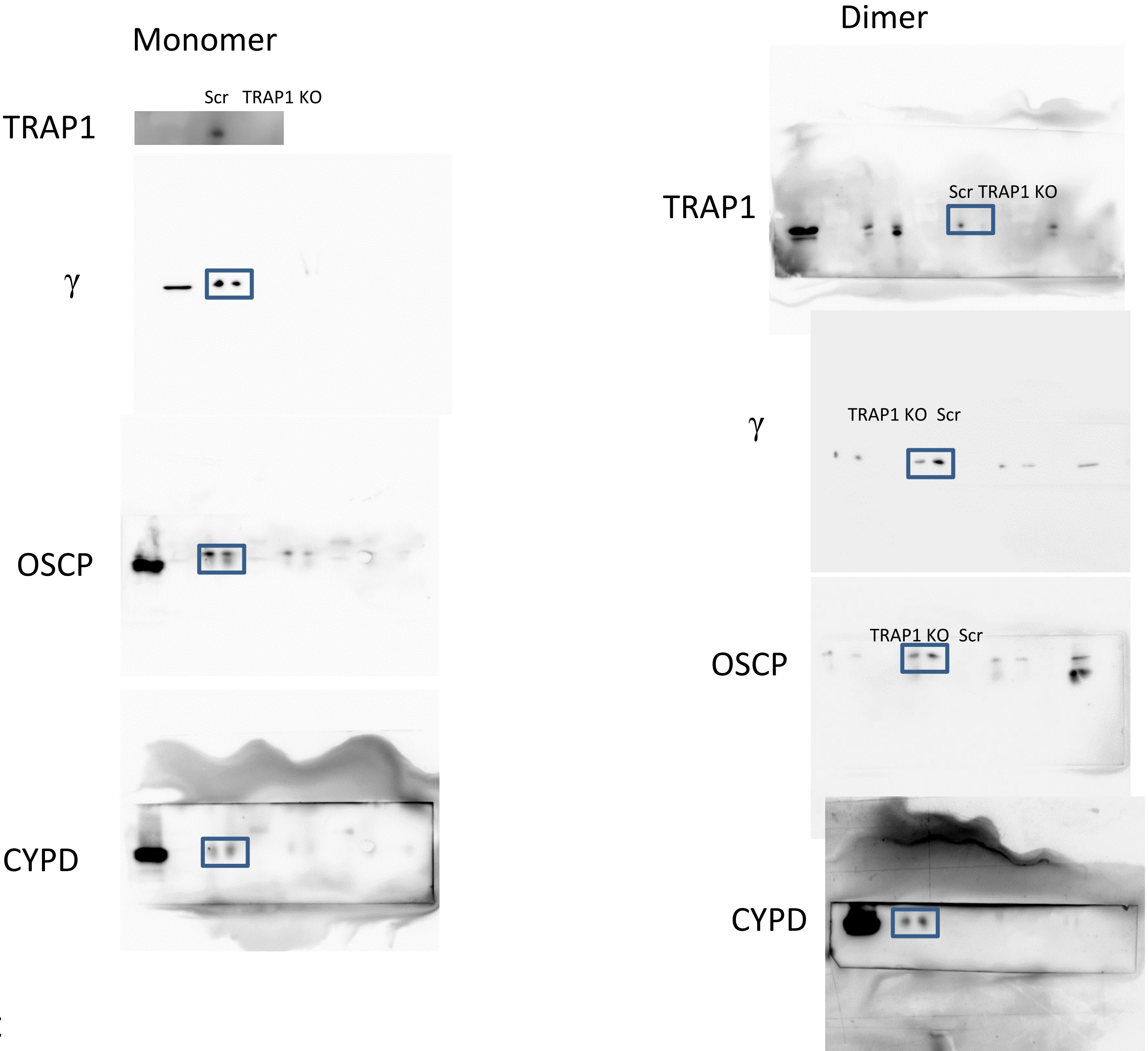
Monomer
Scr TRAP1 KO
TRAP1
γ
OSCP
CYPD
Dimer
TRAP1
Scr TRAP1 KO
γ
TRAP1 KO Scr
OSCP
TRAP1 KO Scr
CYPD

Fig 3 C

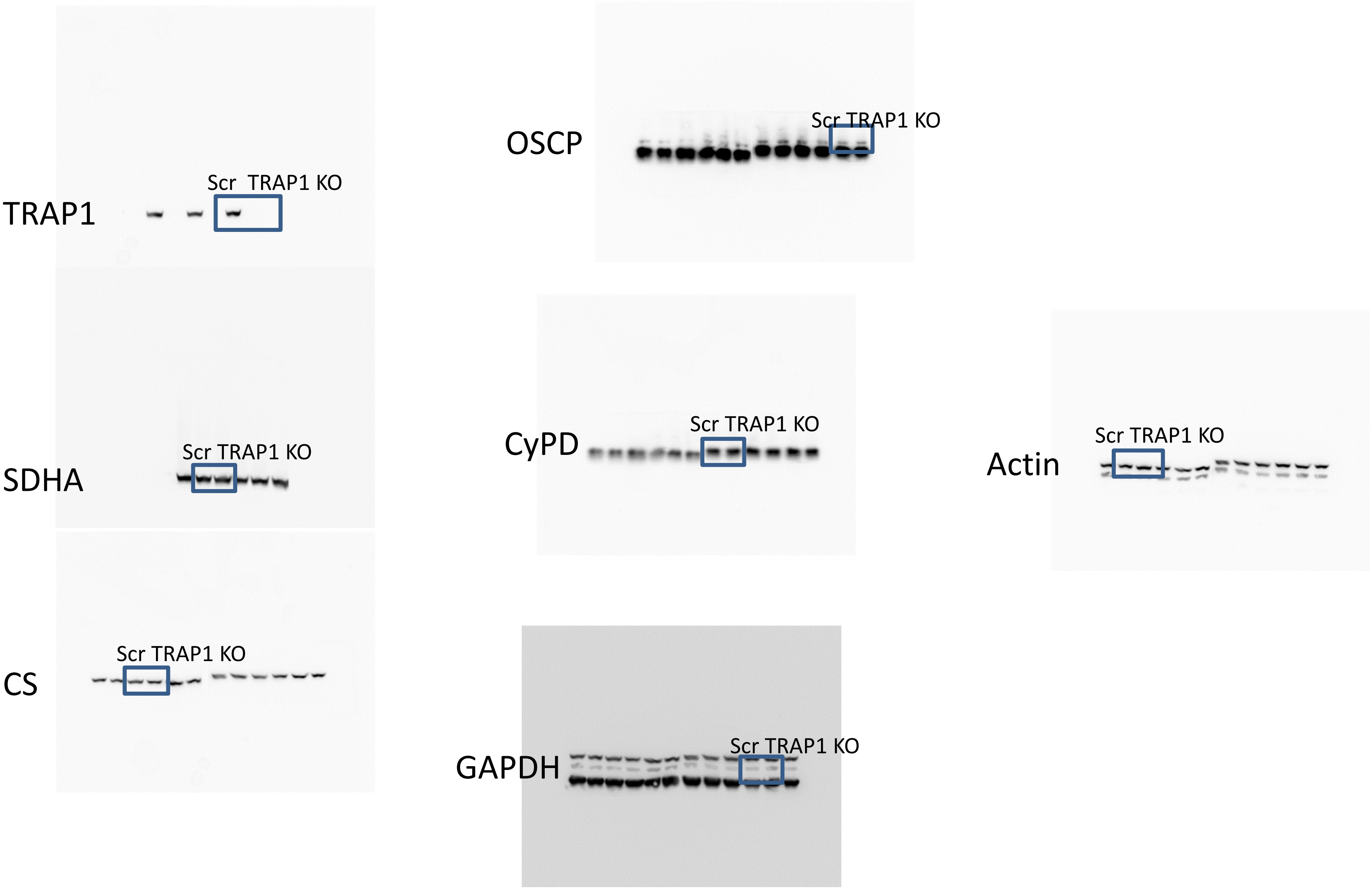
OSCP
Scr TRAP1 KO
TRAP1
Scr TRAP1 KO
SDHA
Scr TRAP1 KO
CyPD
Scr TRAP1 KO
Actin
Scr TRAP1 KO
CS
Scr TRAP1 KO
GAPDH
Scr TRAP1 KO

Fig 3 D

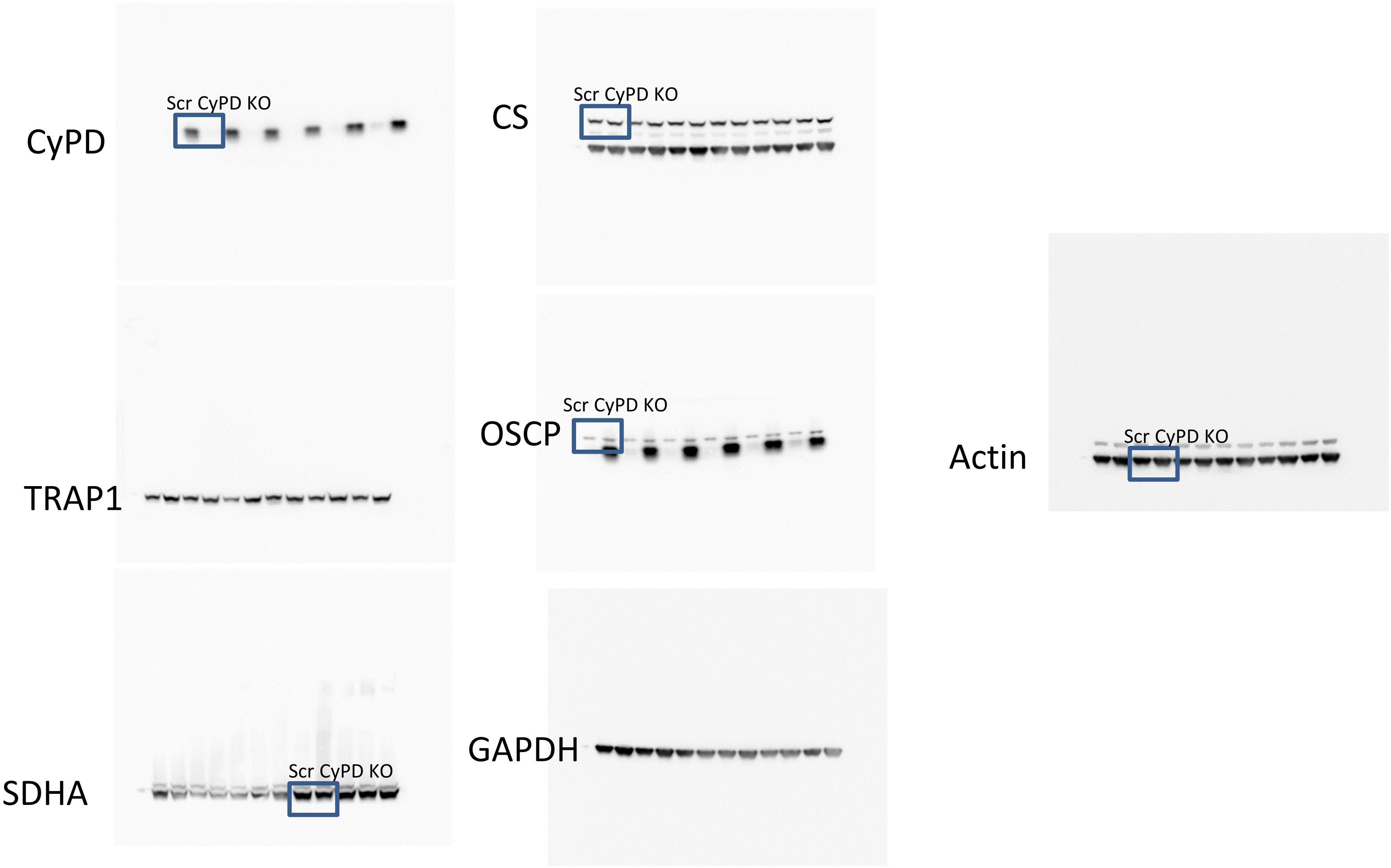
CyPD
Scr CyPD KO
CS
Scr CyPD KO
TRAP1
OSCP
Scr CyPD KO
Actin
Scr CyPD KO
SDHA
Scr CyPD KO
GAPDH

Fig 3F
